# The Integrator complex regulates differential snRNA processing and fate of adult stem cells in the highly regenerative planarian *Schmidtea mediterranea*

**DOI:** 10.1371/journal.pgen.1007828

**Published:** 2018-12-17

**Authors:** David Schmidt, Hanna Reuter, Katja Hüttner, Larissa Ruhe, Franziska Rabert, Florian Seebeck, Manuel Irimia, Jordi Solana, Kerstin Bartscherer

**Affiliations:** 1 Max Planck Institute for Molecular Biomedicine, Münster, Germany; 2 Medical Faculty, University of Münster, Münster, Germany; 3 Centre for Genomic Regulation (CRG), The Barcelona Institute of Science and Technology, Barcelona, Spain; 4 Universitat Pompeu Fabra, Barcelona, Spain; 5 Systems Biology of Gene Regulatory Elements, Berlin Institute for Medical Systems Biology, Max-Delbrück Center for Molecular Medicine, Berlin, Germany; 6 Hubrecht Institute for Developmental Biology and Stem Cell Research, CT Utrecht, The Netherlands; University of Oxford, UNITED KINGDOM

## Abstract

In multicellular organisms, cell type diversity and fate depend on specific sets of transcript isoforms generated by post-transcriptional RNA processing. Here, we used *Schmidtea mediterranea*, a flatworm with extraordinary regenerative abilities and a large pool of adult stem cells, as an *in vivo* model to study the role of Uridyl-rich small nuclear RNAs (UsnRNAs), which participate in multiple RNA processing reactions including splicing, in stem cell regulation. We characterized the planarian UsnRNA repertoire, identified stem cell-enriched variants and obtained strong evidence for an increased rate of UsnRNA 3’-processing in stem cells compared to their differentiated counterparts. Consistently, components of the Integrator complex showed stem cell-enriched expression and their depletion by RNAi disrupted UsnRNA processing resulting in global changes of splicing patterns and reduced processing of histone mRNAs. Interestingly, loss of Integrator complex function disrupted both stem cell maintenance and regeneration of tissues. Our data show that the function of the Integrator complex in UsnRNA 3’-processing is conserved in planarians and essential for maintaining their stem cell pool. We propose that cell type-specific modulation of UsnRNA composition and maturation contributes to *in vivo* cell fate choices, such as stem cell self-renewal in planarians.

## Author summary

Stem cells not only give rise to all specialized cell types of the body through differentiation but also to new stem cells through the process of self-renewal. Modifications of messenger RNAs (mRNAs) following their transcription play an important role in a stem cell’s decision between self-renewal and differentiation. However, since these cells are rare and difficult to access in mammals, the underlying mechanisms have mostly been studied in cultured cells. Here, we employ the planarian flatworm *Schmidtea mediterranea* that maintains a large pool of highly potent stem cells throughout life as an *in vivo* model to analyze the function of UsnRNAs, important regulators of multiple mRNA processing steps, in stem cell regulation. We show that stem cells express more UsnRNA sequence variants compared to differentiated cells. Moreover, the activity of the Integrator complex, which mediates UsnRNA maturation, is increased in stem cells. Depletion of Integrator complex components disrupts their global splicing pattern and affects the processing of histone mRNAs resulting in the loss of stem cell self-renewal and, consequently, in defects of tissue homeostasis and regeneration. Thus, we propose that planarian stem cell self-renewal *in vivo* depends on differential variant expression and efficient processing of UsnRNAs.

## Introduction

The cell types composing a multicellular organism differ significantly in morphology and function, despite their identical genetic information. These features are determined in a differentiation process starting from a common progenitor, a stem cell, through a number of cell fate decisions. While cell fate can be influenced by regulation at every step of gene expression as well as of function and turnover of the gene products, the role of transcriptional changes has been investigated in greatest detail. The regulation of pluripotent stem cell fate, in particular, has been attributed to networks of transcription factors and chromatin modifiers [1–3]. However, recent studies identified key functions of post-transcriptional RNA processing in stem cell self-renewal and fate regulation including alternative splicing (AS) [4–7] and -polyadenylation [8] as well as chemical nucleotide modifications [9]. Intriguingly, core components of the spliceosome, including Uridyl-rich small nuclear RNAs (UsnRNAs), were recently implicated in the regulation of pluripotency [10,11]. The studies uncovering these mechanisms employed mammalian embryonic stem cells (ESCs) or induced pluripotent stem cells (iPSCs) *in vitro*. However, comparable cell types are present *in vivo* only during a short period of development or are exceedingly rare. The role of these factors *in vivo* remains, thus, largely obscure.

In contrast to mammals, some invertebrates maintain highly potent stem cells lifelong. The stem cells of the planarian *S*. *mediterranea*, collectively termed neoblasts, constitute around 25% of all adult body cells and a substantial fraction of them are pluripotent [12]. This abundance of adult stem cells is the basis of the outstanding regenerative abilities and tissue plasticity of adult planarians (reviewed in [13]). The ability to modify neoblast dynamics by tissue amputation or feeding and to transplant them between individual animals, allowing full repopulation of a neoblast-depleted animal from a single donor neoblast, together with their amenability to RNA interference (RNAi) make planarians a powerful model organism for studying stem cell regulation *in vivo*. In a recent study, we identified distinct AS patterns of planarian neoblasts and of their differentiated progeny and showed that they are shaped by the RNA binding protein *bruli*, a CELF family member, and its antagonists of the MBNL family, respectively [14]. Depletion of these factors disturbs not only splicing patterns but also the balance of neoblast self-renewal and differentiation confirming their role in fate regulation. Similarly, a member of the Sm family of proteins, which associate with UsnRNAs forming small nuclear ribonucleoproteins (snRNPs) of the spliceosome, has been implicated in neoblast proliferation [15].

UsnRNAs are short, highly-structured, non-coding RNAs mediating splice site recognition and catalyzing the splicing reaction. In addition to these essential functions as constituents of the splicing machinery, some UsnRNAs regulate splice site choice and alternative polyadenylation [16]. UsnRNA sequence variants are differentially expressed during development [17] and U1 snRNA variants in particular, were shown to control the expression of cell fate regulators [10,18]. Except for U6 and U6atac, UsnRNAs are transcribed by RNA polymerase 2 (RNApol2) but, in contrast to mRNAs, they are not polyadenylated. Instead, the 3’-ends of their primary transcripts are formed by a co-transcriptional cleavage reaction catalyzed by the Integrator complex [19]. This 3’-processing step is required for their nuclear export, their association with Sm proteins and, consequently, for their function in splicing. The Integrator complex consists of at least 14 subunits (*ints*) of which *ints9* and *ints11* form an endonuclease that cleaves nascent pre-UsnRNAs close to a sequence element termed 3’ box [19,20]. By mediating U7 snRNA maturation, the Integrator complex also indirectly facilitates the 3’-processing of replication-dependent histone mRNAs [21,22]. In addition to this canonical function, the complex or a subset thereof was implicated in enhancer RNA (eRNA) processing, pause-release of transcription and the response to DNA damage (reviewed in [23]). Given these important functions, it is not surprising that inactivation of *ints* severely affects development in a range of animal models [24–28].

Here, we analyzed the differential expression and 3’-processing of UsnRNAs, as central components of the mRNA processing machinery, in neoblasts *in vivo*. We characterized the UsnRNA repertoire of *S*. *mediterranea* and identified neoblast-enriched sequence variants. In line with higher expression levels of *ints*, we found evidence for enhanced UsnRNA 3’-processing in neoblasts compared to differentiated cells. Using RNAi-mediated *ints* depletion, as a means of disrupting UsnRNA maturation, we analyzed their role in neoblast fate regulation and identified an essential function in the maintenance of the neoblast pool.

## Results

### Identification of neoblast-enriched planarian UsnRNAs

**Fig 1:**
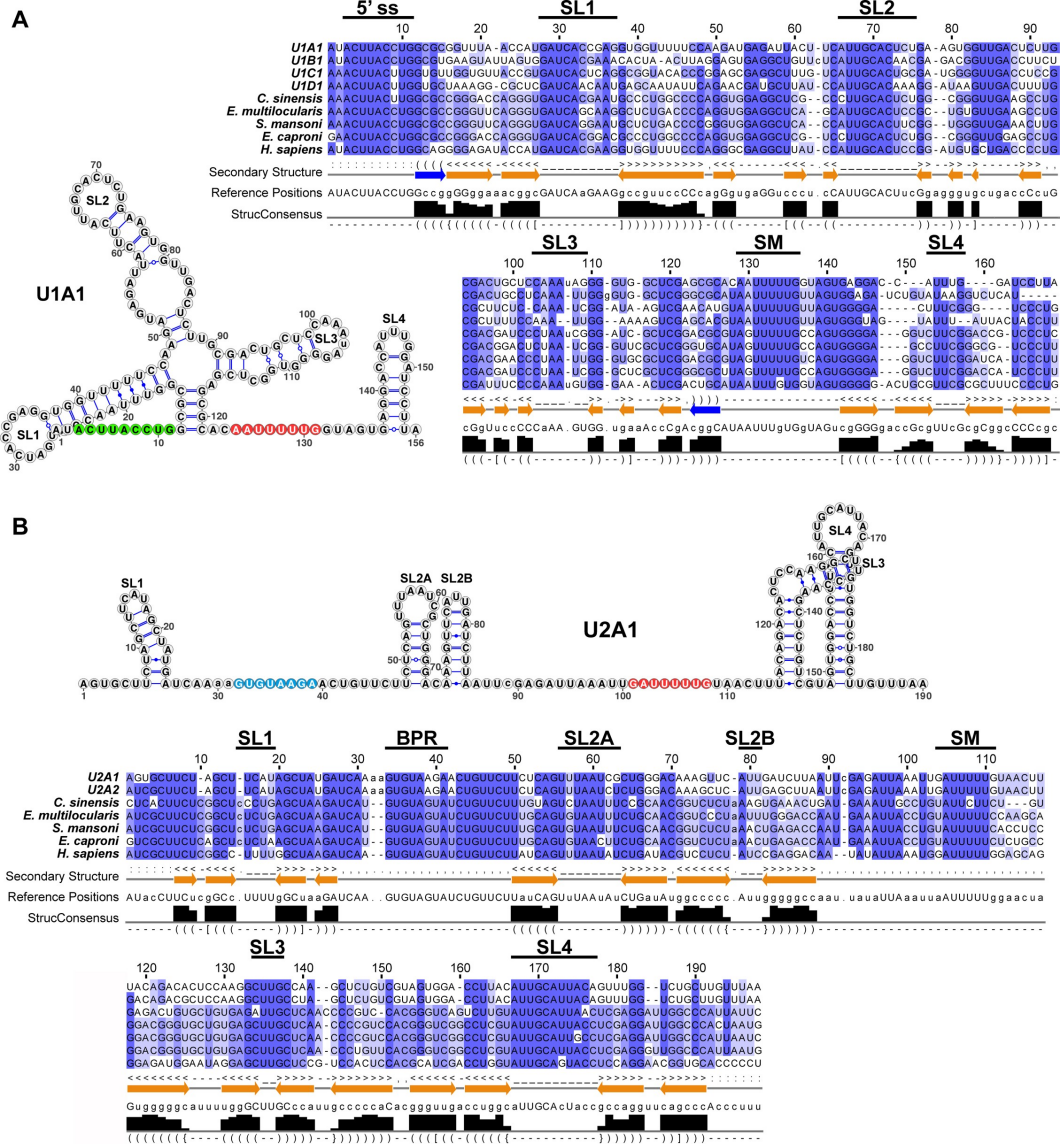
Features of *Schmidtea mediterranea* major spliceosomal UsnRNAs. Representative predicted secondary structures of (A) U1 and (B) U2 snRNAs and sequence alignments to their homologs of other Platyhelminthes (*Clonorchis sinensis*, *Echinococcus multilocularis*, *Schistosoma mansoni* and *Echinostoma caproni*) and *Homo sapiens*. Primary sequence similarity is indicated in violet and secondary structure conservation (StrucConsensus) is shown below the alignments. Evolutionary conserved structural features and functional sequence elements are indicated: 5’ ss = 5’ splice site recognition motif (green); SL = stem loop; SM = SM protein binding site (red); BPR = branch-point recognition sequence (blue).

UsnRNA variants are differentially expressed during development and have been implicated in pluripotency maintenance *in vitro* [10,17,18]. To analyze their function in adult stem cells *in vivo*, we first characterized the *S*. *mediterranea* UsnRNA repertoire employing a combination of *in silico* identification of putative UsnRNA genes and validation of their expression by small RNA sequencing. Since UsnRNA secondary structures are evolutionary conserved, we performed a structure-guided similarity search for candidate genes in the *S*. *mediterranea* genome [29–31]. This search identified a total of 217 spliceosomal UsnRNA candidates, of which 55 met the statistical threshold (S1 Table). Small RNA sequencing validated the expression of 22 of them. Among these were multiple variants of U1 (11), U2 (2), U4 (3) and U6 (2) snRNA, many of which were present as multiple copies in the genome. Additionally, we identified a putative U4atac candidate, which did not meet the statistical threshold but was validated by RT-PCR (S1 Table, S1A Fig). The predicted secondary structures of the identified UsnRNA types and sequence alignment to their orthologs of other Platyhelminthes species and of *Homo sapiens* showed substantial conservation of both structural and functional sequence elements, such as the splice site recognition motifs of U1, U5, U6 and U11 snRNA, the branch point recognition motif of U2 and U12 snRNA as well as the sequences mediating the dimerization of U4- and U6-type snRNAs (Fig 1, S1C and S1D Fig, S2 Fig). As expected, the Sm protein binding site (PuA(U)_5-6_Pu) required for incorporation into snRNP particles is present in all Sm-class UsnRNAs and is highly similar to its vertebrate counterpart [32,33]. Altogether, our analysis identified *S*. *mediterranea* orthologs for all spliceosomal UsnRNA types (S1B Fig).

**Fig 2:**
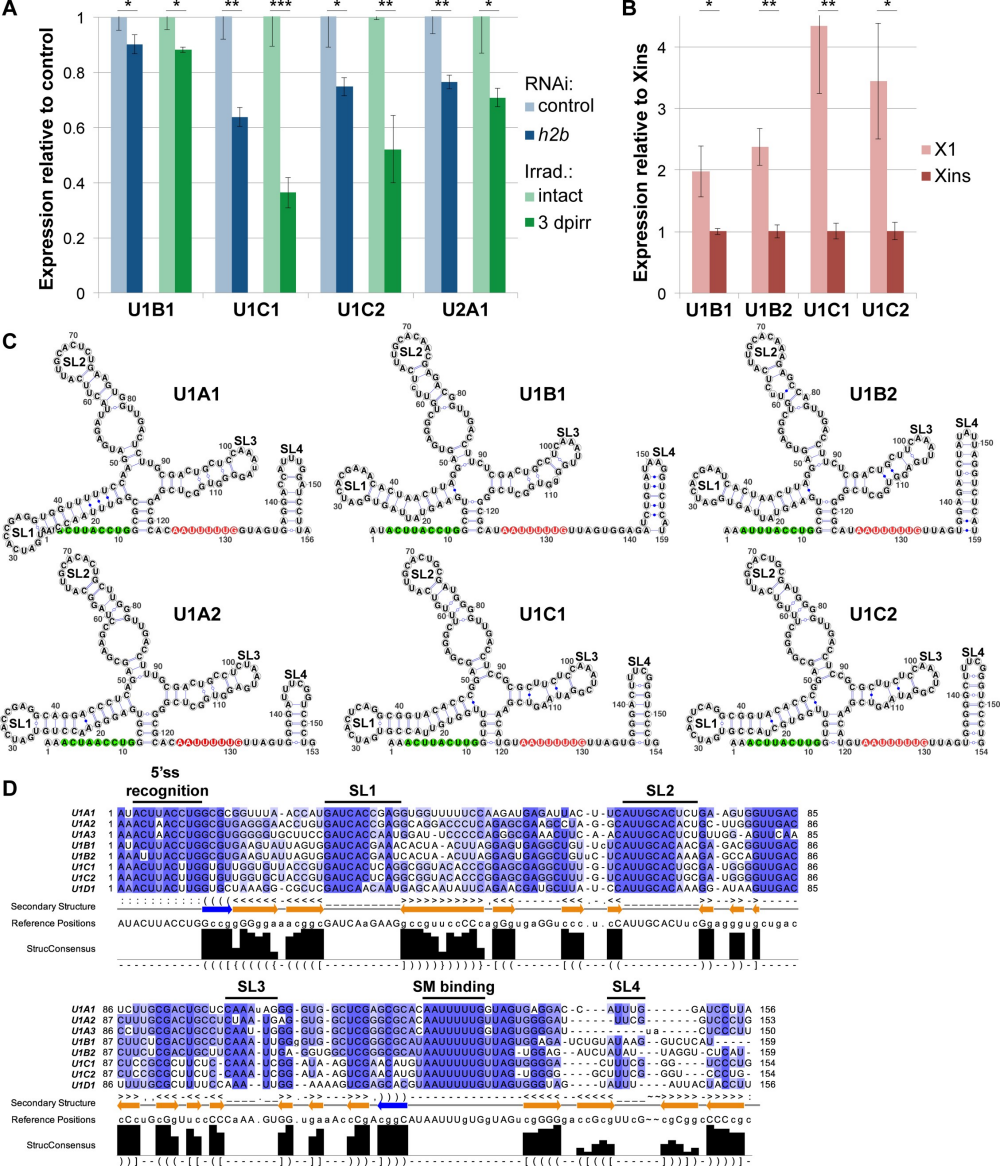
Neoblast-enriched expression of variant U1 snRNAs. (A, B) UsnRNA variant expression, quantified by qRT-PCR, in animals depleted of neoblasts by *h2b* RNAi (blue) or lethal irradiation (green) and in cell populations isolated by FACS (red). Shown are the variants with significantly changed expression relative to the respective control. Results for other variants are shown in S3 Fig. Expression levels are the averages of three biological replicates, normalized to those of *gapdh* (error bars represent standard deviation; two-sided t-test, * p<0.05, ** p<0.01, *** p<0.001). (C) Predicted secondary structures and (D) sequence alignment of *S*. *mediterranea* U1 snRNA variants. Evolutionary conserved structural features and functional sequence elements are indicated: 5’ ss = 5’ splice site recognition motif (green); SL = stem loop; SM = SM protein binding site (red). Primary sequence similarity is indicated in violet and secondary structure conservation (StrucConsensus) is shown below the alignments.

To test whether UsnRNA variants are differentially regulated between neoblasts and differentiated cells, we compared their expression levels in intact planarians with those in neoblast-depleted ones, by qRT-PCR. We employed *h2b* RNAi [34] and γ-irradiation [35] as two independent and efficient means of neoblast eradication. Interestingly, while the majority of the identified UsnRNAs were expressed uniformly (S3A Fig), three U1- and one U2 snRNA variants were significantly reduced in both stem cell-depletion conditions (Fig 2A). U1C1, U1C2 and U2A1 showed the most prominent reduction (23–63%), which suggests a substantial enrichment in neoblasts. We corroborated these findings by comparing UsnRNA variant expression between cell populations isolated by fluorescence-activated cell sorting (FACS). Dissociated cells were sorted according to their size and DNA content into a fraction of small cells with high DNA content (>2n) consisting mainly of neoblasts in G2/M phase of the cell cycle (X1 population) and a fraction of larger cells with 2n DNA content, comprised mainly of differentiated cells (Xins population) [36]. Whereas both variants of U1B and U1C snRNA were significantly enriched in the X1 compared to the Xins cell fraction (2–4 fold, Fig 2B), the mild enrichment of U2A1 snRNA in X1 cells (13% higher than in Xins cells, S3B Fig) was not statistically significant. While the predicted secondary structures of the neoblast-enriched variants are overall very similar to those of the uniformly expressed U1A snRNAs (Fig 2C), sequence alignment revealed base changes in both 5’ splice site recognition motif and in loop sequences mediating the interactions with RNPs (Fig 2D). Notably, U1C1 and U1C2, the U1 snRNA variants with the strongest neoblast enrichment, possess an alternative base within the 5’ splice site recognition motif (Fig 2D, S1 Table, S3C Fig) raising the possibility that these UsnRNAs may play a specialized role, perhaps in the recognition of specific splice sites.

### Disruption of UsnRNA maturation through depletion of Integrator complex subunits

To evaluate the role of the identified UsnRNA variants in planarians, we employed dsRNA-mediated RNA interference (RNAi). However, in contrast to planarian mRNAs [37], UsnRNAs could not be efficiently depleted using this approach (S4A Fig). As functional maturation of most UsnRNAs requires a 3’ cleavage reaction catalyzed by the Integrator complex [19,38], depletion of Integrator complex subunits (*ints*) by RNAi should constitute an efficient means for inducing a UsnRNA loss-of-function condition in planarians [19,22]. We performed BLAST searches for *ints* in the *S*. *mediterranea* transcriptome [39] and identified potential homologs for 12 out of 14 known subunits (S2 Table). For *Smed*-*ints5* and -*ints10*, homologous candidates with low sequence similarity were identified using the Planmine database BLAST annotation. Based on their BLAST scores and their previous identification in an *S*. *mediterranea* stem cell proteome [40], we focused on *Smed*-*ints3* and -*ints9* (*ints3 and ints9*) for further analysis.

**Fig 3:**
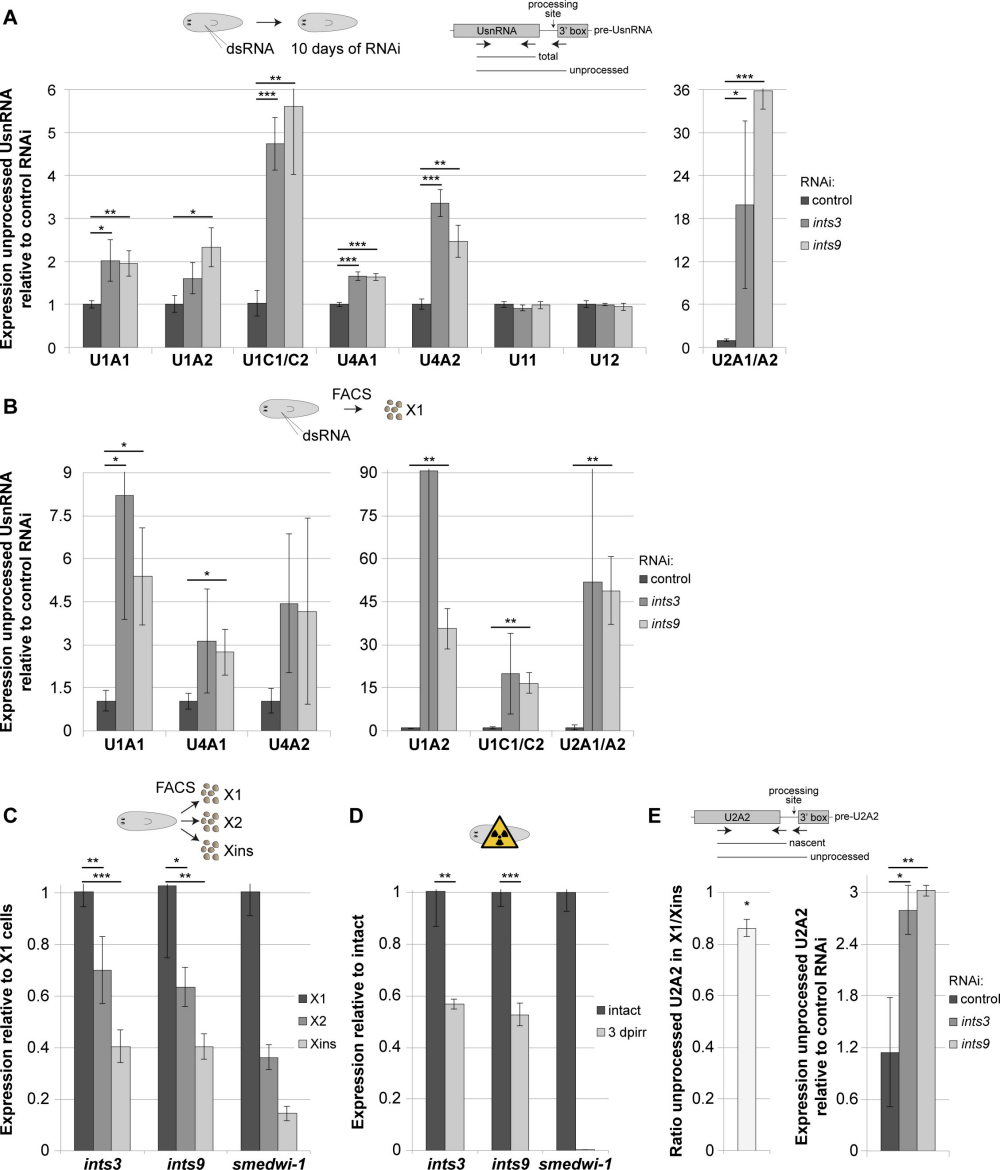
Expression of *integrator* genes is increased in neoblasts and is essential for UsnRNA processing. qRT-PCR quantification of transcript levels. (A, B) Depletion of Integrator complex subunits results in a significant accumulation of 3’-unprocessed UsnRNAs in both intact animals and in the neoblast-enriched X1 FACS fraction. (C, D) *ints3* and *ints9* mRNA levels were significantly higher in the X1 fraction compared to both the X2 and the Xins fraction, and were strongly down-regulated in γ-irradiated animals depleted of neoblasts. (E) 3’-unprocessed U2A2 snRNA, relative to the total nascent U2A2 level, was less abundant in RNA of X1 cells compared to that of Xins cells (left chart; average of the X1/Xins ratios of 3 biological replicates; p = 0.02, two-sided heteroscedastic t-test) and accumulated substantially in X1 cells upon *ints* RNAi (right chart). Expression levels are the averages of three biological replicates normalized to those of the total pool of the respective UsnRNA variant in A+B, to those of *gapdh* in C+D and to those of total nascent U2A2 in E (error bars represent standard deviation; two-sided t-test, * p<0.05, ** p<0.01, *** p<0.001).

To test for a requirement of the Integrator complex for UsnRNA 3’-processing in planarians, we inhibited *ints3* or *ints9* expression by RNAi, followed by qRT-PCR quantification of 3’-unprocessed UsnRNAs. To correct for potential effects of *ints* RNAi on UsnRNA transcription, we normalized the level of the 3’-unprocessed UsnRNA to that of the respective total pool. Importantly, depletion of *ints3* or *ints9* resulted in a significant accumulation of the unprocessed forms of all tested UsnRNA variants, except for U11 and U12 (Fig 3A). We observed the highest accumulation of the unprocessed form for U2 snRNAs (20- and 35-fold in *ints3*- and *ints9* RNAi animals, respectively). These results show that *ints3* and *ints9* are functional planarian homologs of Integrator complex subunits and that their depletion disrupts UsnRNA maturation.

### High *ints* expression and Integrator-mediated UsnRNA processing activity in neoblasts

Next, we employed the UsnRNA processing assays to RNA of X1 cells isolated by FACS from *ints* RNAi animals. For all UsnRNAs analyzed, we observed even higher average accumulation of the unprocessed forms in *ints* RNAi X1 cells than for RNA extracted from entire *ints* RNAi animals. Except for U4A2, all unprocessed forms were significantly increased in either *ints9* or *ints3* RNAi X1 cells, or both, compared to X1 cells of control RNAi animals (Fig 3B).

The requirement of the Integrator complex for UsnRNA 3’-processing in X1 cells tempted us to analyze the expression of *ints* in different cell populations. Thus, we compared the mRNA levels of the putative *ints* homologs in previously published RNA expression data of FACS-isolated cell fractions [41]. Interestingly, all of the putative *ints* homologs showed higher expression in the X1 FACS fraction than in the Xins fraction, with an average enrichment of 4.8-fold, and five of the subunits had been previously classified as neoblast-enriched [41] (S3 Table). We confirmed the significantly higher expression of *ints3* and *ints9* in the X1 compared with the Xins population by qRT-PCR (Fig 3C). Consistently, γ-irradiated animals showed strongly reduced levels of both transcripts (Fig 3D).

Increased mRNA expression of *ints* may translate into a higher activity of the snRNA 3’-processing machinery. We therefore quantified the levels of 3’-unprocessed UsnRNA in X1 and Xins cell fractions isolated by FACS. To be able to correct for differential snRNA transcription- and degradation rates in the different cell fractions, we developed a qRT-PCR assay that quantifies the total nascent transcript level by detecting a short 3’ extension present in 3’-unprocessed UsnRNA and in an Integrator-processed, short-lived intermediate but not in the mature UsnRNA. We focused on U2A2 snRNA since the Integrator-processed intermediate of its counterpart in other species (U2+10 nt) was reported to have a relatively long 3’-extension [42,43], allowing to distinguish it from the mature form by PCR (Scheme in Fig 3E). Sanger sequencing identified position 187 as the mature end of U2A2 snRNA (S4B Fig) and we employed a reverse primer annealing to positions 165–192 to quantify the total nascent transcript level. After normalizing to this level, we found that the 3’-unprocessed U2A2 snRNA was, on average, 14% less abundant in RNA of the X1 compared to the Xins fraction (p = 0.02, two-sided heteroscedastic t-test), indicating that neoblasts possess not only a distinct composition of spliceosomal UsnRNAs but likely also a higher UsnRNA processing activity than differentiated cells. Importantly, both *ints3 and ints9* RNAi resulted in an approximately 3-fold increase in the level of unprocessed U2A2 snRNA in the X1 population (Fig 3E), demonstrating the importance of the Integrator complex for UsnRNA processing in neoblasts.

### Neoblasts are progressively lost upon *ints* depletion

**Fig 4:**
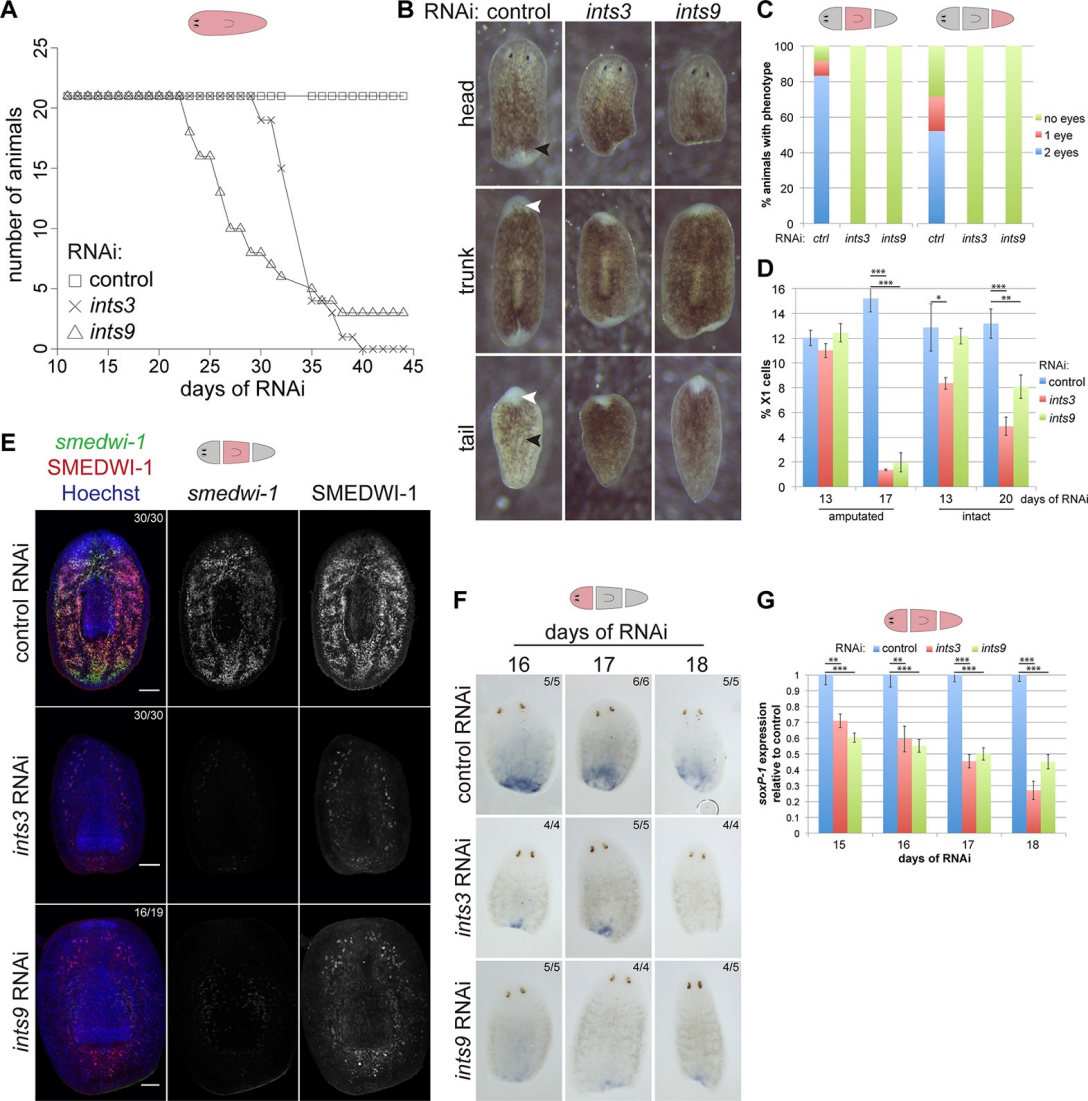
*ints* depletion disrupts neoblast maintenance as well as tissue homeostasis and regeneration. (A) Survival curves of homeostatic *ints* RNAi animals showing median survival times of 32 and 26 days after the first RNAi treatment (days of RNAi) for *ints3* and *ints9* RNAi, respectively. Corresponding live images are in S4A Fig. (B) Representative live images of *ints* RNAi fragments 7 days post amputation (7 dpa; 20 days RNAi) displaying significantly reduced blastema size, lack of eye regeneration (white arrowheads) and defective remodeling of existing tissues to form a pharynx (black arrowheads). (C) Quantification of eye regeneration in trunk and tail fragments corresponding to B (n>32; 2 independent experiments). (D) Cytometry analysis of dissociated cells derived from *ints* and control RNAi animals revealing a progressive loss of the X1 populations in both amputated and intact *ints* RNAi animals. Shown is the average proportion of X1 cells in all gated cells of 3 biological replicates. (E) FISH and immunostaining of *smedwi-1* mRNA and SMEDWI-1 protein, respectively, in trunk fragments at 7 dpa (20 days of RNAi) confirming the *ints* RNAi-induced loss of neoblasts and their immediate progeny. The proportion of trunk fragments with the depicted phenotype are indicated (2 independent experiments; Scale bar = 100 μm). (F+G) WISH staining- and qRT-PCR time-course (2–5 dpa) showing that the expression of *soxP-1*, a marker of sigma neoblasts, is strongly reduced in *ints* RNAi fragments. The expression levels are the averages of three biological replicates normalized to those of *gapdh*. (two-sided t-test, * p<0.05, ** p<0.01, *** p<0.001). Error bars represent standard deviation.

Neoblasts are strictly required for tissue turnover and regeneration in planarians [13]. Hence, an essential role of Integrator complex-mediated UsnRNA processing in neoblast function would be reflected by defects in these processes upon *ints* depletion. Monitoring intact *ints* RNAi animals for several weeks, we found that they developed head regression and ventral curling, phenotypes indicative of a neoblast defect [44], and died after a median survival time of 27 days (*ints9* RNAi) or 33 days (*ints3* RNAi) after the first dsRNA injection (Fig 4A, S5A Fig). Interestingly, amputated *ints* RNAi animals initiated regeneration but developed only a very small regeneration blastema and completely failed to regenerate photoreceptors or pharynges (Fig 4B). 7 days following amputation, when 1 or both eyes were already visible in the majority of control trunk and tail fragments, none of the *ints* RNAi fragments had regenerated eyes (Fig 4C). Like homeostatic RNAi animals, they eventually underwent ventral curling and died approximately 3 weeks after the first RNAi treatment.

To test whether *ints* depletion indeed affects the neoblast population, we performed flow cytometry of cell suspensions prepared from regenerating *ints* RNAi vs. control RNAi animals and observed a strong reduction of the X1 fraction at 7 days post amputation (dpa; 17 days of RNAi; Fig 4D). Interestingly, while we observed a strong effect of *ints* RNAi on UsnRNA processing in neoblasts already at 13 days of RNAi (Fig 3B), the proportion of X1 cells at that time-point was still comparable to that of control RNAi animals. This demonstrates that neoblasts are progressively lost during regeneration of *ints* RNAi animals and suggests that the disruption of UsnRNA processing is an indirect cause of this loss. To exclude that the neoblast loss is merely a consequence of the observed regeneration defect, we performed the same analysis on homeostatic *ints* RNAi animals, which showed a similar, progressive reduction of the neoblast fraction (Fig 4D). In line with a slower turnover of neoblasts during homeostasis, their proportion decreased at a slower rate in homeostatic compared to regenerating *ints* RNAi animals.

We next confirmed the observed neoblast loss using Fluorescent In Situ Hybridization (FISH) of *smedwi-1* mRNA, the prototypical stem cell marker in planarians [44], which was strongly diminished in regenerating *ints3* and *ints9* RNAi animals at 20 days of RNAi (Fig 4E). Immunofluorescence co-staining of the SMEDWI-1 protein, which is maintained in the immediate progeny of neoblasts (*smedwi-1*^-^/SMEDWI-1^+^ cells), revealed that in both *ints* RNAi conditions these cells were strongly reduced, albeit to a lesser extent than neoblasts, likely as a consequence of defective neoblast maintenance [45] (Fig 4E). Depletion of a third putative Integrator complex subunit, *Smed*-*ints11*, pheno-copied the regeneration defects and neoblast loss observed upon *ints3* and *ints9* RNAi (S5B Fig).

Recent studies identified neoblast subpopulations with different lineage potential [46]. To determine whether *ints* depletion affects sigma neoblasts, which are collectively pluripotent and therefore the most potent subtype known, we analyzed the expression of *soxP-1*, a marker of these cells, by Whole-mount In Situ Hybridization (WISH) and qRT-PCR at different times following amputation. Strikingly, the sigma neoblast population was already substantially reduced at 2 dpa (15 days of RNAi) in both *ints* RNAi conditions and decreased further during the following days (Fig 4F and 4G; S6 Fig), suggestive of a progressive loss of sigma neoblasts in *ints* RNAi animals. Together with increased *ints* expression and lower levels of unprocessed U2A2 snRNA in neoblasts, these results may suggest a function of UsnRNA processing in the maintenance of the neoblast pool.

### *ints* RNAi affects 3’-processing of histone mRNAs and disrupts neoblast splicing patterns

**Fig 5:**
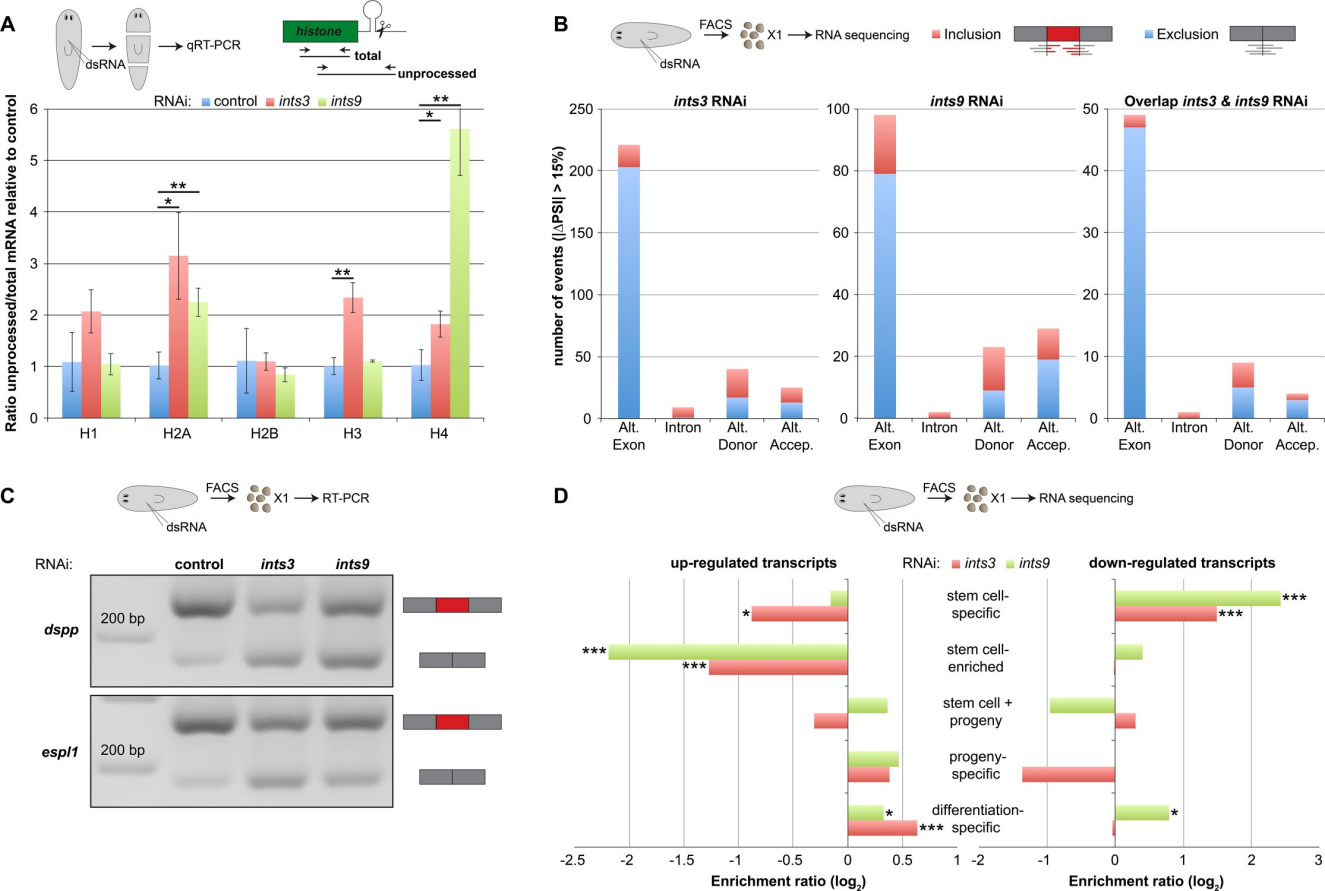
*ints* RNAi interferes with *histone* mRNA processing and disrupts the splicing- and gene expression profiles of neoblasts. (A) qRT-PCR quantification showing that the 3’-unprocessed mRNAs of H2A, H3 and H4 accumulate in regenerating *ints* RNAi animals (3 dpa; 16 days of RNAi). Expression levels are the averages of three biological replicates normalized to those of the total pool of the respective histone (Error bars represent standard deviation; two-sided t-test, * p<0.05, ** p<0.01). (B) Overview of the splicing events significantly changed in neoblasts depleted of *ints3*, *ints9* or in both conditions showing that the exclusion of alternative (Alt.) exons (exon skipping) is the primary splicing defect induced by *ints* RNAi. PSI = Percent Spliced-In. For details see S4 Table. (C) Two examples of *ints* RNAi-induced exon skipping events in neoblasts affecting transcripts encoding putative homologs of *dspp* and *espl1*, analyzed by RT-PCR. Results for all other RT-PCR validations are shown in S7A Fig. (D) Comparison of the gene expression changes induced by *ints* RNAi in X1 cells to the transcripts differentially expressed between the X1, X2 and Xin FACS fractions [41]. Transcripts annotated as stem cell-specific were statistically significantly over-represented among the transcripts down-regulated in *ints* RNAi X1 cells (threshold for differentially expressed genes in *ints* RNAi neoblasts: p_adj_<0.05; |log_2_ fold-change| > 1; Statistical test for over-/under-representation: hypergeometric test, * p<0.05, *** p<0.001).

Disruption of UsnRNA maturation may affect neoblast maintenance by altering different UsnRNA-mediated molecular processes. U7 snRNA, for instance, is required for histone mRNA 3’-end processing [21,22] and histone expression was implicated in neoblast maintenance [34,45]. Hence, we tested the effect of *ints* depletion on planarian histone processing. We designed qRT-PCR assays to quantify the 3’-unprocessed and the total pool of 5 canonical histone transcripts in RNA of *ints-* and control RNAi animals at 3 dpa (16 days of RNAi). Normalizing to the total transcript levels, we observed a significant accumulation of 3’-unprocessed mRNAs of histones H2A and H4 for both *ints* RNAi conditions as well as of H3 for *ints3* RNAi animals (Fig 5A), indicating that the known function of the Integrator complex in U7 snRNA- and, thus, indirectly in histone mRNA-processing is conserved in planarians.

Given the key function of UsnRNAs in splicing, we hypothesized that the *ints* RNAi-induced loss of neoblasts is, at least in part, due to the disruption of their splicing patterns. To globally characterize the effects of *ints* depletion on the splicing patterns of neoblasts, we sequenced the transcriptomes of X1 cells isolated from *ints3*- and *ints9* RNAi animals at 3 dpa (16 days of RNAi) and compared their splicing patterns to that of control RNAi X1 cells. As described recently, we quantified the inclusion frequency (PSI; Percent Spliced In) of alternative exons and introns as well as the differential usage of alternative donor and acceptor splice sites [14,47,48]. This analysis identified 295 and 152 splicing events that showed a significant change in inclusion levels (│PSI *ints* RNAi-PSI ctrl RNAi│>15%, see Methods) in X1 cells of *ints3* and *ints9* RNAi animals, respectively, compared to their control RNAi counterparts (Fig 5B, S4 Table). Although the total number of splicing changes differed, a strong bias towards exon skipping (exclusion of exons) was observed for both RNAi conditions (68.8% and 51.9% of all events, respectively) and only a very small number of intron retention events were detected (2.7% and 1.3%, respectively). Moreover, a core set of 63 splicing events were affected in the same direction in both RNAi conditions (S4 Table). 14/15 (93%) tested exon skipping events shared by *ints3* and *ints9* RNAi X1 cells were validated by RT-PCR (Fig 5C, S7A Fig). These results suggest that the defects in UsnRNA maturation induced by *ints* RNAi affect primarily the exon definition step of splicing in neoblasts. Remarkably, a comprehensive analysis of sequence and structural features of exons skipped upon either RNAi treatment revealed that these are very AT-rich (S8D Fig).

We analyzed the sequencing data of isolated X1 cells further for *ints* RNAi-induced changes of gene expression, which identified a total of 1351 or 477 transcripts differentially expressed between *ints3* or *ints9* RNAi and the control, respectively (p_adj_<0.05, │log_2_ fold-change│>1, S5 Table). Importantly, the overlap between the mis-spliced and the differentially expressed transcripts was minimal (15 and 0 for *ints3* and *ints9* RNAi, respectively; S7B Fig), suggesting that the majority of the splicing defects did not directly affect mRNA stability. The overlap between *ints3* and *ints9* RNAi-induced changes in transcript levels, however, was substantial (S7C Fig) indicating that these changes are indeed caused by a loss of Integrator complex function. We next compared these changes to expression data of Labbé *et al*. [41], who analyzed the transcriptomes of FACS sorted cell fractions. Interestingly, among the transcripts down-regulated in *ints3* or *ints9* RNAi X1 cells, we observed a highly significant over-representation of those classified as stem cell-specific (X1) by Labbé *et al*. (Fig 5D, S5 Table). While these expression changes affected only a subset of all stem cell-specific transcripts, they may indicate a disruption of the normal neoblast gene expression pattern.

Together, these data suggest that defects in histone mRNA processing and splicing collectively contribute to the neoblast loss observed after *ints* RNAi.

### *ints* RNAi disrupts neoblast self-renewal

**Fig 6:**
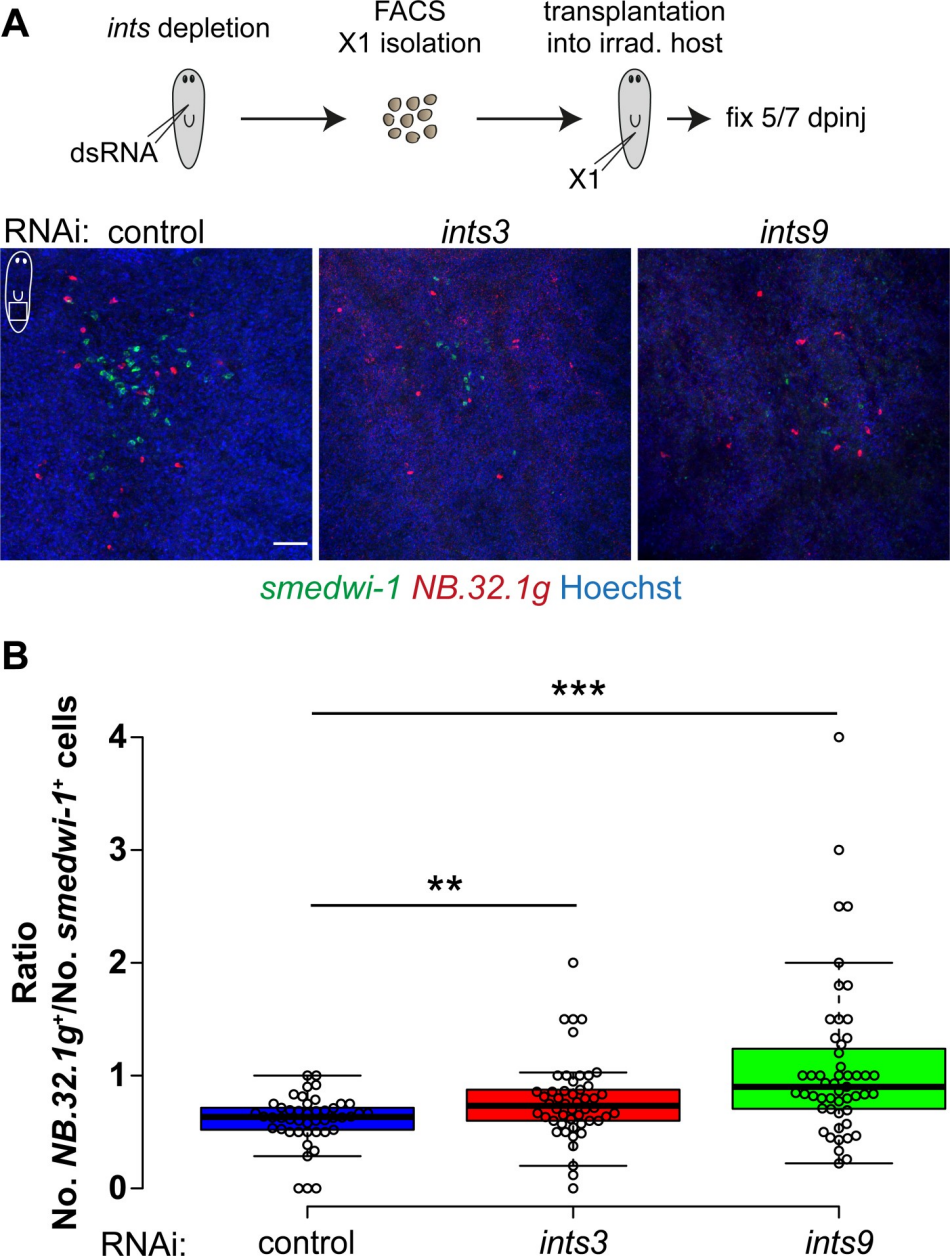
*ints* depletion disrupts neoblast self-renewal. (A) Neoblast transplantation assay. FACS-isolated X1 cells of RNAi animals were transplanted into neoblast-depleted, wild-type hosts. The cell type composition of colonies derived from transplanted neoblasts was analyzed by FISH staining of the neoblast- and the early progeny marker *smedwi-1* and *NB*.*32*.*1g*, respectively. *ints*-depleted neoblasts produced smaller colonies containing an increased proportion of early progeny. Scale bar = 50 μm. dpinj = days post neoblast injection. (B) Quantification of colony composition showing a significant increase in the proportion of early progeny (*NB*.*32*.*1g*^+^) cells generated from *ints*-depleted neoblasts. Data of three independent experiments. Circles represent individual colonies. Center lines show the medians; box limits indicate the 25th and 75th percentiles as determined by R software; whiskers extend 1.5 times the interquartile range from the 25th and 75th percentiles, outliers are represented by dots. n = 46, 50, 51 colonies. Two-sided t-test: ** p<0.01, *** p<0.001.

We next analyzed the effects of *ints* depletion on the self-renewal of neoblasts. Since the high density of these cells and of early neoblast progeny complicates their quantification directly within the RNAi animals, we transplanted FACS-isolated X1 cells of *ints* or control RNAi animals into neoblast-depleted wild-type hosts and analyzed the resulting colonies 5 and 7 days after transplantation (Fig 6A). We identified neoblasts and their differentiating progeny by FISH staining of *smedwi-1* and *NB*.*32*.*1g* [35], respectively, and quantified their proportions. As shown in Fig 6A and 6B, the colonies derived from *ints3* and *ints9*-depleted neoblasts were smaller than those derived from control RNAi neoblasts and contained a significantly higher proportion of differentiating *NB*.*32*.*1g*^+^ cells (p<0.01 and p<0.001, respectively), demonstrating that *ints* depletion disrupts the ability of neoblasts to self-renew and changes their fate towards differentiation.

**Fig 7:**
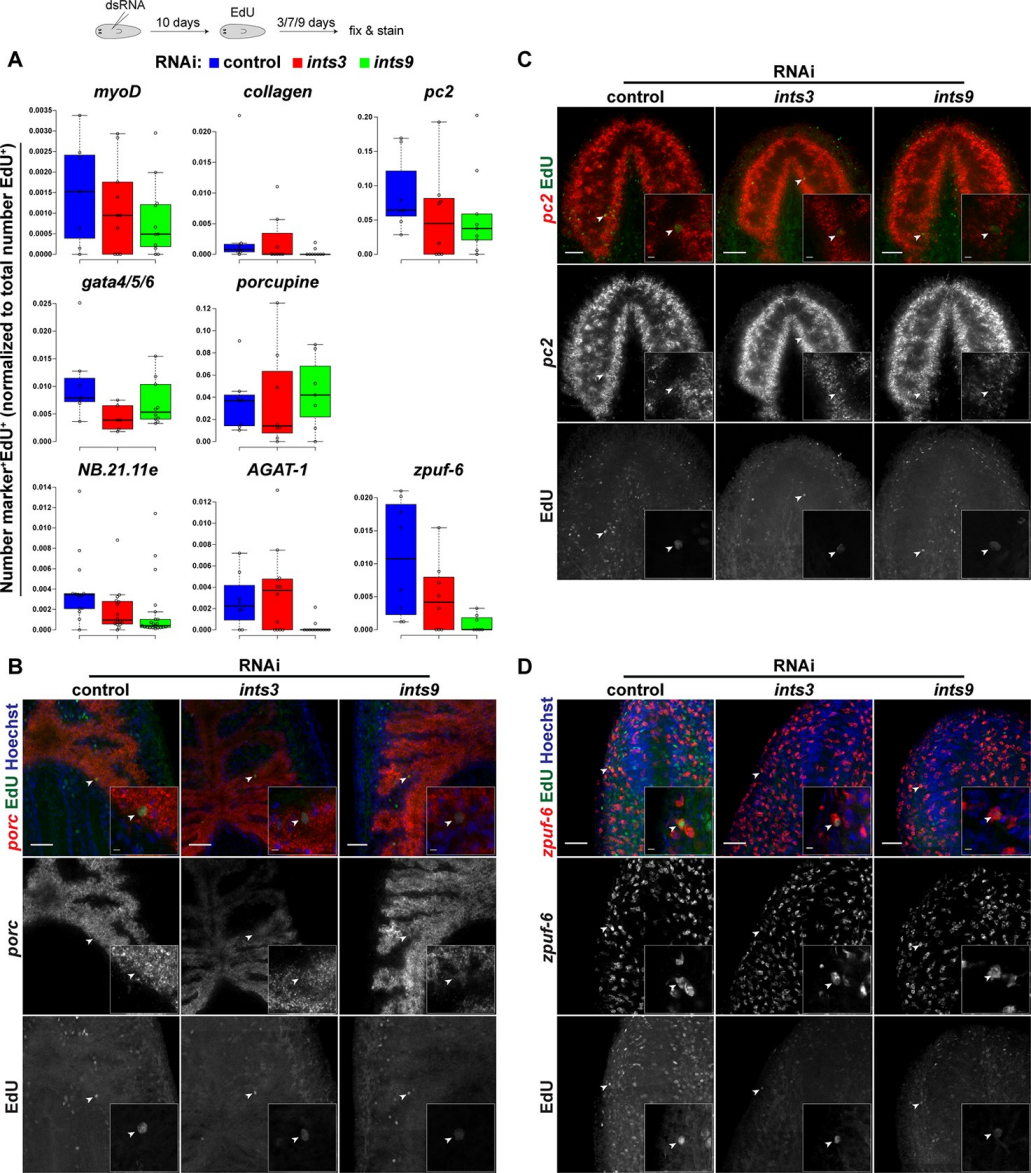
*ints* RNAi reduces differentiated cell output. EdU pulse-chase labeling combined with FISH staining of differentiation markers. 10 days following the first dsRNA injection, animals were pulsed with EdU, fixed 3, 7 or 9 days later and cell type markers were visualized by FISH. (A) Quantification of EdU-positive cells expressing the indicated marker, normalized to the total number of EdU-positive cells. Chase periods were 3 days for *NB*.*21*.*11*.*e*, *myoD* and *gata4/5/6*, 7 days for *AGAT-1* and 9 days for *porcupine*, *pc2*, *zpuf-6* and *collagen*. Circles represent measurements of individual animals. Center lines show the medians; box limits indicate the 25th and 75th percentiles as determined by R software; whiskers extend 1.5 times the interquartile range from the 25th and 75th percentiles, outliers are represented by dots. (B, C, D) Representative images of EdU-labelled cells expressing the intestinal (*porcupine*), neuronal (*pc2*) or epidermal progeny (*zpuf-6*) marker. Overview images and insets are single confocal sections acquired with a 20x and a 63x objective, respectively. Arrowheads indicate marker^+^EdU^+^ cells. Scale bars: overview pictures = 50 μm, magnifications = 5 μm.

To test the capacity of *ints*-depleted neoblasts to differentiate, we pulse-labelled proliferating cells with the thymidine-analog Ethynyl-deoxyuridine (EdU) and analyzed their progeny after different chase periods for the expression of cell type markers representing all germ layers by FISH staining. We normalized the number of marker^+^EdU^+^ cells to the total number of EdU^+^ cells, to account for the substantially lower number of EdU-incorporating neoblasts of *ints* RNAi animals (Fig 7A). The median numbers of EdU-positive cells expressing early (*NB*.*21*.*11e*^+^), intermediate (*AGAT-1*^+^) [35] and late (*zpuf-6*^+^) [49] markers associated with the epidermal lineage were substantially lower in *ints9-*depleted animals compared with controls. A similar reduction was observed for *myoD*^+^EdU^+^ muscle cell progenitors [50] and *collagen*^+^EdU^+^ muscle cells [51]. The numbers of *gata*4/5/6^+^EdU^+^ gut precursors [12] and of terminally-differentiated intestinal cells expressing *porcupine* [52] were reduced in *ints3* RNAi animals. Furthermore, we observed a mild reduction in the number of EdU^+^ neurons expressing *prohormone convertase 2* (*pc2*) [53] in both *ints* RNAi conditions. Importantly, EdU-positive cells expressing markers of late progeny and of terminally differentiated cell types were still observed in *ints* RNAi animals (Fig 7B–7D). These findings indicate that neoblast progeny is able to differentiate even at very low Integrator complex activity, yet gives rise to a lower number of differentiated cells, possibly due to a reduction in general cell fitness.

## Discussion

Here, we describe the UsnRNA repertoire of the planarian *S*. *mediterranea* and investigate the impact of disturbed Integrator complex-mediated UsnRNA processing on the fate of its stem cells. We first identified spliceosomal UsnRNAs using a bioinformatics approach that has been successfully employed in other non-mammalian model organisms [30,54,55]. The validation of their expression by small RNA sequencing effectively removed pseudogenes, as shown independently by RT-PCR. While this approach might have excluded some UsnRNA candidates that are expressed at low levels and/or have high sequence similarity to other candidates, the numbers of validated genes encoding the different UsnRNA types are in the range of those predicted for their counterparts in the closely-related Schistosomes [56,57] suggesting that they represent the majority of the *S*. *mediterranea* UsnRNA repertoire.

As was recently reported for U1 snRNA in cultured mammalian stem cells [10,18], our analysis showed that neoblasts express higher levels of several U1 snRNA variants when compared to differentiated cell types. The similarity of their predicted structure and the conservation of functional sequence motifs suggests that these variants could function in splicing. Given that neoblasts also express several spliceosome components at higher levels [14] and that increased spliceosome activity has been linked to the maintenance of pluripotency [11], we speculate that increased UsnRNA levels support a higher spliceosome activity. Moreover, the modified 5’ splice site recognition motif present in the neoblast-enriched U1C1 and U1C2 snRNA variants indicates that they might facilitate the use of specific 5’ splice sites or, alternatively, act as dominant negative regulators suppressing differentiation-related AS events. A role for these variants in alternative polyadenylation is also plausible [16]. Of note, even minor U1 snRNA variants may exert substantial effects on gene expression [18].

In FACS-sorted cell fractions enriched for neoblasts, we found lower levels of 3’-unprocessed U2A2 snRNA, relative to the total nascent transcript, than in cell fractions consisting of differentiated cells. This raises the possibility that the 3’-processing of UsnRNAs constitutes a cell type-dependent control mechanism for UsnRNA abundance and hence for UsnRNA-dependent processes, such as splicing. While lower levels of unprocessed UsnRNAs could alternatively be explained by higher degradation rates, the observed increase of unprocessed U2A2 in neoblasts upon inhibition of the UsnRNA 3’-processing machinery supports our hypothesis. Together with our qRT-PCR results and previously published RNAseq data [41], which show that *ints*-encoding transcripts are enriched in neoblasts, it is likely that this increased processing activity is, at least in part, due to higher *ints* expression in these cells.

Testing the function of UsnRNAs directly requires efficient and highly sequence-specific methods for UsnRNA depletion or over-expression, both of which are currently not available in *S*. *mediterranea*. In contrast to mRNAs, UsnRNAs could not depleted by dsRNA-mediated RNAi, which is likely due to differences in expression levels, subcellular localization or structure of these RNA types. We therefore used RNAi against Integrator complex subunits as efficient means to disrupt UsnRNA maturation and hence function. This caused a plethora of splicing defects and the accumulation of unprocessed histone mRNAs. The latter is likely a consequence of defective U7 snRNA processing by the Integrator complex, since U7 snRNA is known to mediate the maturation of replication-dependent histone mRNAs [21,22,58]. Given the importance of histone expression for neoblast maintenance [34], the reduced processing of H2A, H4 and H3 mRNAs is likely to contribute to the neoblast maintenance defect of *ints* RNAi animals. While splicing defects are an expected consequence of the loss of UsnRNA processing, the very high proportion of exon skipping, but not intron retention, events among the splicing changes observed in both *ints* RNAi conditions was surprising and suggests that *ints* depletion impairs primarily exon definition. Interestingly, very similar splicing changes were observed in *ints8*-mutant mammalian cells [59], substantiating our results and supporting an evolutionary conserved function of the Integrator complex in facilitating splicing fidelity. As many of the exon skipping events are likely to disrupt the reading frame of the corresponding transcripts, reducing the production of functional proteins and causing the accumulation of potentially cytotoxic proteins, they may interfere with neoblast maintenance. Indeed, depletion of either *ints3* or *ints9* disrupted tissue homeostasis and regeneration, likely as a consequence of the gradual neoblast loss. While the Integrator complex has not been implicated in stem cell maintenance to date, knock-out of *ints* genes causes early developmental lethality in diverse animal models that could well be explained by defects of stem cell maintenance [24–27].

Investigating a putative effect of *ints* RNAi on the transcriptional profile of neoblasts we revealed a considerable de-regulation of gene expression. Whether this was due to defective histone processing or the disruption of a putative transcriptional regulator function of the Integrator complex [60–62] is unclear but the minimal overlap between mis-spliced and differentially expressed transcripts suggest that defective splicing is not a major contributor. Despite a stronger effect of *ints9* RNAi on the survival of planarians, we observed a higher number of both mis-spliced and differentially expressed transcripts in *ints3-* compared to *ints9* RNAi neoblasts, which may be due to differences in knock-down kinetics or efficiency. Neoblast-specific transcripts were strongly over-represented among those down-regulated in both *ints3* and *ints9*-depleted neoblasts. It is important to point out that these transcriptomic changes occur in neoblasts and, thus, do not represent changes in cell type composition. However, they might be early indicators of a neoblast fate change. Transplantation of *ints* RNAi neoblasts into irradiated host animals confirmed the fate change of these cells towards differentiation. Importantly, since the recipient animals in this experiment were not injected with dsRNA, this cell fate change is likely not due to a loss of Integrator complex function in the host tissue, but rather represents a cell-autonomous phenotype of the transplanted cells. In line with the considerably smaller colonies derived from transplanted *ints* RNAi neoblasts, EdU pulse-chase experiments revealed that the production of a number of progenitor and differentiated cell types is reduced upon *ints* depletion. Since the number of neoblasts initially labelled with EdU cannot be determined in these experiments, it is unclear whether the reduced proliferation of *ints*-depleted neoblasts contributes to this lower cell output. Nonetheless, the presence of EdU-labelled *pc2*^+^, *porcupine*^+^ and *zpuf-6*^+^ cells in the brain, the gut and underneath the epidermis of *ints*-depleted animals, respectively, suggests that the low levels of *ints* expression remaining after the incomplete knock-down achieved by RNAi allows for differentiation, at least of a portion of the neoblast progeny.

**Fig 8:**
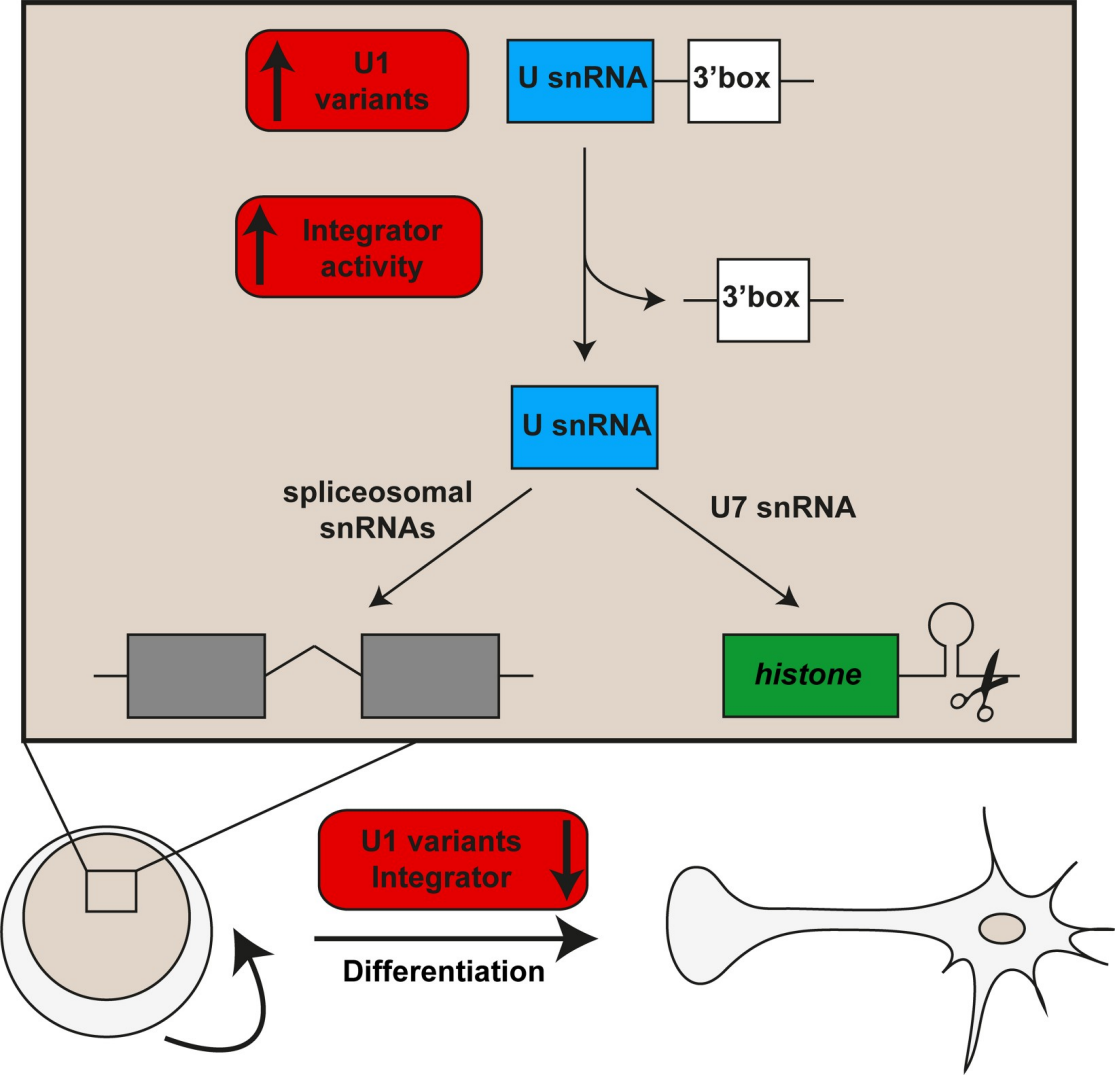
Model of the role of UsnRNAs in neoblast maintenance. Neoblasts express higher levels of U1 snRNA variants and have higher UsnRNA 3’-processing activity, likely due to the high expression levels of Integrator complex components. Together with increased expression of spliceosomal proteins [14], this may result in a modified specificity and enhanced activity of the spliceosome in neoblasts compared to differentiated cell types. In addition, an increased activity of the Integrator complex may allow efficient histone mRNA 3’-processing through U7 snRNA maturation. Together, these features likely contribute to neoblast maintenance by generating a specific set of protein isoforms as well as by enabling the high turnover of mRNAs and proteins in these mitotically active cells.

Neoblasts, similar to germ line stem cells in other organisms, are highly dependent on post-transcriptional regulators of mRNA localization, splicing and/or translation [14,15,58,63–66] as well as on factors generating regulatory RNAs [44,67]. Our study now extends this list by the Integrator complex, which appears to have a dual function in the post-transcriptional regulation of transcripts important for neoblasts: By processing UsnRNA 3’-ends, it mediates splicing and histone mRNA maturation, both of which were previously linked to neoblast maintenance [14,15,34,45]. We propose a model (Fig 8) in which the increased expression of U1 snRNA variants together with enhanced, Integrator-mediated UsnRNA maturation facilitate a higher activity and/or modified specificity of neoblast spliceosomes as well as the efficient U7-dependent processing of histone mRNAs. This may contribute to neoblast maintenance by generating a specific set of protein isoforms as well as by enabling the high turnover of mRNAs and proteins in these mitotically active cells.

## Materials and methods

Unless specified otherwise, reagents and devices were purchased from Thermo Fisher Scientific and were used according to the manufacturers recommendations.

### UsnRNA prediction, structure modelling and classification

UsnRNA multiple sequence alignments annotated with consensus secondary structures were obtained from the Rfam database v12.0 [31]. For each spliceosomal UsnRNA type, a covariance model was built, calibrated and then searched against the genome of the asexual *Schmidtea mediterranea* biotype (Asxl v1.1) [29] using the INFERNAL 1.1.1 programs cmbuild, cmcalibrate and cmsearch [30], respectively, with default settings. The identified candidate sequences were aligned to the respective covariance model using cmalign of INFERNAL and the resulting, predicted secondary structures were visualized using VARNA 3.93 [68]. For U6atac structure prediction, the candidate sequence was extended at the 3’-end by adding 32 nucleotides from the genomic sequence to fit the model length. Multiple sequence alignments generated by cmalign were visualized with Jalview 2.10.4b1 [69]. Phylograms were generated using Clustal Omega [70]. Variants of the same UsnRNA type were clustered according to sequence similarity and the clusters were named in alphabetical order, starting from the one with the highest number of uniquely-mapping RNAseq reads. Within clusters, variants were given consecutive numbers according to best-mapping read count. Sequence logos were generated using WebLogo generator [71].

### Planarian husbandry, RNAi and EdU pulse-chase

Animals of the asexual, clonal *Schmidtea mediterranea* line BCN-10 (originally provided by E. Salo), were maintained as described [72] and treated as specified in S7 Table. Double-stranded RNA (dsRNA) was transcribed from PCR amplicons using T7 RNA polymerase as described previously [73]. Control RNAi animals were injected with dsRNA targeting a region of the green fluorescent protein (GFP) coding sequence. For regeneration experiments, animals were amputated pre- and postpharyngeally into head, trunk and tail fragments. Neoblast depletion by *h2b* RNAi was performed as described previously [34]. For lethal γ-irradiation, animals were exposed to a dose of 60 Gy using a Gammacell 40 Exactor (Nordion). For EdU pulsing, animals were incubated in (2′S)-2′-Deoxy-2′-fluoro-5-ethynyluridine (F-ara-EdU, Sigma Aldrich) [74] dissolved in planarian artificial medium (PAM) containing 3% (v/v) DMSO for 16 hours. F-ara-EdU concentrations of 0.4, 0.2 and 0.1 mg/ml were used for 3, 7 and 9 day chase, respectively. Animals were maintained in the dark in PAM containing 50 μg/ml gentamycin throughout the chase period and then fixed with formaldehyde [75], subjected to FISH and then EdU was stained using the EdU DetectPro Imaging Kit 647 (baseclick GmbH) according to the manufacturer’s protocol.

### RNA isolation, cDNA synthesis and qRT-PCR

RNA was isolated using the TRIzol or TRIzol LS reagent. RNA for qRT-PCR analysis of UsnRNAs or for mRNA sequencing was treated with TURBO DNase followed by either phenol-chloroform extraction and ethanol precipitation or by purification on NucleoSpin RNA XS columns (Macherey-Nagel). For the latter, RNA was adjusted to a final ethanol concentration of 53% before loading onto the column to improve recovery of small RNA species. First strand cDNA was transcribed using the RevertAid H Minus First Strand cDNA Synthesis Kit with random hexamers (for UsnRNA and histone mRNA assays) or oligo dT primers (for all other mRNA assays), respectively. For the analysis of unprocessed UsnRNA in wild-type X1 and Xins fractions, RNA prepared as described above was subjected to whole transcriptome amplification using the Ovation qPCR system (NuGEN), which was performed with 45 ng of RNA in technical duplicates according to the manufacturers protocol and then pooled. qRT-PCR analysis was performed on the Real-Time PCR System 7500 or 9100 (Applied Biosystems) using either the Universal Probe Library (Roche) together with TaqMan Universal PCR Master Mix or the iTaq universal SYBR Green supermix (Bio-Rad). Primers and probes are summarized in S6 Table. Relative expression levels were calculated as previously described [76]. 3’-unprocessed U2A2 levels were normalized to those of the nascent transcript. End-point RT-PCR was performed in a Mastercycler pro (Eppendorf) using Quick-Load Taq 2X Master Mix (NEB) and analyzed by agarose gel electrophoresis. For S6A Fig, the intensity of the gel bands was quantified using Fiji [77].

### Small RNA sequencing

RNA species of 50–500 nucleotides were isolated from total RNA of intact, wild-type animals by electrophoresis on a 7% polyacrylamide gel containing 7M Urea, extracted from the homogenized gel piece in gel extraction buffer (300mM sodium acetate, 1mM EDTA, 0.01 U/μl SUPERase• In RNase Inhibitor) at 4°C overnight, filtered through a Spin-X centrifuge tube filter (Corning) and concentrated by ethanol precipitation. 500 ng of size-selected RNA was de-capped using RNA 5’ pyrophosphohydrolase (NEB) according to the manufacturers recommendations and then concentrated by ethanol precipitation. The de-capped RNA was then subjected to sequencing library preparation using the NEBNext Small RNA Library Prep Set for Illumina (NEB) according to the manufacturers protocol and analyzed on an Illumina HiScan SQ. Using Bowtie v1.1.2 [78], the RNAseq reads were mapped onto a compilation of all UsnRNA candidate sequences identified by Infernal cmsearch, each extended by 25 nucleotides on the 3’ end to facilitate the mapping of pre-snRNA-derived reads. To account for the high sequence similarity between variants of the same UsnRNA type, a dual mapping strategy was employed by counting either only uniquely-mapping reads (for validating candidate gene expression) or all best mapping reads (for estimating expression levels). The Bowtie parameters for reporting best- or unique alignments were -a—best -v2 or -a -m1 -v2, respectively. To quantify uniquely-mapping reads, identical candidate sequences in the compilation (colour-coded in S1 Table) were represented by a single sequence. The number of mapped reads were quantified using HTseq-count [79]. For Sanger sequencing (GATC Biotech), individual UsnRNAs were PCR-amplified from the sequencing library described above using a forward primer specific for the respective UsnRNA and the SR RT Primer of the NEBNext Small RNA Library Prep Set for Illumina as the reverse primer (S6 Table).

### Flow cytometry

Flow cytometry was performed as described previously [80], except that Hoechst 33342 was used at 10 μg/ml and cells were stained at 24°C.

### Fluorescence-activated cell sorting (FACS) and cell transplantation

X1-cell isolation was performed on a FACSAria Cell Sorter with its respective software (BD Biosciences) using the protocol described in [12]. Cell transplantations were performed 10 days after the first dsRNA injection as described in [40], except that approximately 3750 X1 cells in 100 nl were injected per host animal. 5 and 7 days after cell injection, animals were formaldehyde-fixed and processed as described below (S7 Table). For subsequent RNA isolation from cell fractions, cells were stained as described above for flow cytometry and sorted directly into TRIzol LS reagent.

### Global analysis of splicing patterns and of differential gene expression

cDNA libraries were prepared from total RNA of the X1 FACS-fraction in biological triplicates using the TruSeq Stranded mRNA Library Prep Kit (Illumina) according to the manufacturers recommendations. Paired-end sequencing (2x75 cycles) was performed on an Illumina NextSeq500. Analysis of alternative splicing was performed using vast-tools v2.0.2. (https://github.com/vastgroup/vast-tools), as recently described [14,47]. Differentially spliced AS events were identified using vast-tools compare, with the following parameters:—min_dPSI 15—min_range 5—noVLOW—p_IR—paired (i.e. │average ΔPSI│>15 and a minimum pairwise difference between paired replicates of | ΔPSI | > 5 in the same direction, excluding events with read coverage score VLOW or lower, and filtering introns that did not pass the binomial test for read balance at the exon-intron junctions). S4 Table contains a summary of the splicing analysis results.

For differential expression (DE) analysis, RNAseq reads were mapped to the reference transcriptome (http://vastdb.crg.eu/libs/vastdb.sme.16.02.18.tar.gz) using vast-tools [47] to obtain raw read counts. DEseq2 [81] was then used for read count normalization using size factors and pairwise-comparison using contrasts. Genes with p_adj_<0.05 and log_2_ fold-change ≤ -1 or ≥ 1 were considered significantly differentially expressed. For comparison to previously published RNA expression data of FACS-isolated cell fractions [41], the corresponding transcripts in the to_Smed_v2 transcriptome were identified by BLAST search and the classification of the highest scoring hit was used for enrichment analysis. The enrichment ratio of class X among DE transcripts was calculated according to: (No. DE transcripts with class X / Total No. DE transcripts) / (No. transcripts with class X in background / Total No. transcripts in background), where background are all transcripts evaluated for DE. The significance of the enrichment was determined using a one-sided hypergeometric test. Venn-diagrams representing overlaps were generated using the VennDiagram package in R [82].

### Immunostaining and In Situ Hybridization of animal whole-mounts

Immunostaining was performed as described previously [73] with an anti-SMEDWI-1 antibody (1:1000), generated as described in [83], and Alexa Fluor-conjugated goat-anti-rabbit IgG secondary antibodies (1:400). Chromogenic whole-mount (WISH)- and fluorescent *in situ* hybridizations (FISH) were performed using the protocols described in [75,84–86] and [87], respectively. RNA probes were *in vitro*-transcribed, using T7 or SP6 RNA polymerase, from PCR amplicons generated with the primers listed in S6 Table and labelled with digoxigenin (DIG RNA Labeling Mix, Roche) or dinitrophenol (Label IT Nucleic Acid Labeling Kit, DNP, Mirus Bio). Hoechst 33342 was used at 5 μg/ml as a nuclear counterstain and animals were mounted using Aqua Poly/Mount (Polysciences).

### Image acquisition and analysis, Figure preparation

Bands on agarose gels were visualized using a GEL IX 20 (intas). Live animals and WISH stainings were imaged using a Leica M80 and M165 FC microscope, respectively. Confocal microscopy was performed on a Zeiss LSM700 using the ZEN software. CellProfiler [88] was used for the automated identification and quantification of EdU^+^ and marker^+^EdU^+^ cells. The Classifier functionality of CellProfiler Analyst [89] was applied to the CellProfiler output to improve identification accuracy for *NB*.*21*.*11*.*e*, *MyoD*, *gata4/5/6*, *AGAT-1*, *zpuf-6* and *collagen*-positive cells. Images were processed using Fiji [77] or Adobe Photoshop. Box-Whisker plots were generated using the BoxPlotR web-tool (http://shiny.chemgrid.org/boxplotr/). Figures were prepared in Adobe Illustrator.

### Accession numbers

Next generation sequencing data is available under Bioproject PRJNA397855: SAMN07490271 (X1_gfp_RNAi), SAMN07490272 (X1_ints3_RNAi), SAMN07490273 (X1_ints9_RNAi), SAMN07490274 (small RNAs_wt_intact). Partial coding sequences of *ints* were deposited in Genbank: MF671814 (*ints3*) and MF671815 (*ints9*).

## Supporting information

S1 Fig Validation, classification and sequence features of UsnRNAs. (A) Validation of UsnRNA expression. RT-PCR of all tested UsnRNAs produced bands of the expected size (M: molecular weight marker; 100 & 200 bp). Corresponding reactions without reverse transcriptase (-RT), which control for amplification from genomic DNA, had no or a significantly weaker band. (B) Clustering of validated UsnRNAs based on sequence similarity. # indicates the number of identical copies identified in the *S*. *mediterranea* genome [29]. (C+D) U4, U4atac and U6atac snRNA predicted secondary structures and sequence alignments to known homologs of other Platyhelminthes (*Clonorchis sinensis*, *Schistosoma mansoni* and *Echinostoma caproni*) and *Homo sapiens*. Primary sequence similarity is indicated in violet and secondary structure conservation (StrucConsensus) is shown below the alignments. Evolutionary conserved functional sequence elements are indicated: SM = SM protein binding site (red); U4-U6 or U4atac-U6atac base-pairing (yellow).
(PDF)

S2 Fig Features of *Schmidtea mediterranea* spliceosomal UsnRNAs. Representative predicted secondary structures and sequence alignments to their homologs of other Platyhelminthes (*Clonorchis sinensis*, *Echinococcus multilocularis*, *Schistosoma mansoni* and *Echinostoma caproni*) and *Homo sapiens*. Primary sequence similarity is indicated in violet and secondary structure conservation (StrucConsensus) is shown below the alignments. Evolutionary conserved functional sequence elements are indicated: ss = splice site recognition motif (green); SM = SM protein binding site (red); BPR = branch-point recognition sequence (blue); UsnRNA base-pairing sequence (yellow).
(PDF)

S3 Fig Expression of UsnRNAs in neoblast-depleted planarians and FACS cell fractions. (A+B) UsnRNA variant expression in animals depleted of neoblasts by *h2b* RNAi (blue), by lethal irradiation (green) and in cell fractions isolated by FACS (red), quantified by qRT-PCR. Expression levels are the averages of three biological replicates, normalized to those of *gapdh* (two-sided t-test, * p<0.05, ** p<0.01, *** p<0.001). (C) Sequence logos of the 5’ splice site recognition motif of different U1 snRNA variants. The U1 variants with a high average neoblast-enrichment have an alternative base (C → U) annealing to the -1 position of the 5’splice site.
(PDF)

S4 Fig UsnRNA knock-down and determination of the mature end of U2A2 snRNA. (A) Injection of dsRNAs targeting U1C or U2A snRNAs did not reduce their expression after 14 days of RNAi. Expression levels are the average of at least 3 biological replicates, normalized to those of *gapdh* (two-sided t-test, *** p<0.001). (B) Sanger sequence trace showing the 3’ end of U2A2 and the adapter of the RNAseq library from which it was amplified. The mature end is also indicated in the secondary structure model of U2A2 below.
(PDF)

S5 Fig RNAi phenotypes of *ints3*, *ints9 and ints11*. (A) Live microscopy images showing homeostatic RNAi animals at the indicated times from the first RNAi treatment (days of RNAi). *ints* RNAi animals developed head regression, ventral curling and eventually lysed. (n = 21, 2 independent experiments). (B) Depletion of the putative *ints11* homolog pheno-copied the regeneration and neoblast maintenance defects induced by *ints3* and *ints9* RNAi. All images and data are from trunk fragments at 8 dpa (n > 10 for live images, 21 d RNAi, 1 experiment). Scale bar = 100 μm.
(TIF)

S6 Fig qRT-PCR time-course of neoblast subclass markers in amputated *ints* RNAi animals. The zeta and sigma neoblast subclass markers were described in [46]. The expression levels are the averages of three biological replicates normalized to those of *gapdh*. (two-sided t-test, * p<0.05, ** p<0.01, *** p<0.001).
(PDF)

S7 Fig Validation of splicing defects and overlap of splicing and differential expression data. (A) Results of the RT-PCR validation of 15 alternative exon splicing events identified by RNAseq of *ints* RNAi X1 cells. The intensity of the gel bands (see Fig 4B for an example) representing the exon-included and the exon-excluded form were quantified and used to calculate the PSI value. 14 of the 15 events tested were significantly de-regulated in *ints3* and *ints9* RNAi X1 cells. The events IDs correspond to those in S4 Table (SmeEX(0)nYYYY → ExYYYY). (B, C) Venn-Diagrams showing that the vast majority of the detected splicing defects do not significantly affect the expression of the respective transcript and that a substantial number of gene expression changes are shared by *ints3* and *ints9* RNAi X1 cells. Thresholds for inclusion are the same as in Fig 5B and 5C.
(PDF)

S8 Fig Features of exons affected by *ints* RNAi. (A, B) Prediction of upstream and downstream splice site strength. (C) Determination of exon length. (D) GC content in affected exons. (E, F) Median length of down- and up-stream introns of affected exons. (G) Statistical significance (p-values) for analyses shown in A-F tested by Mann-Whitney-U-test. **AS NC** alternatively spliced, but not affected by *ints* RNAi; **CR** “cryptic” exons (PSI < 5); **CS** random subset of 1000 constitutively spliced exons; **EX** exons with ΔPSI < -15 in *ints vs* control RNAi; **INC** exons with ΔPSI > 15 in *ints* vs control RNAi; bp basepair; ss splice site
(PDF)

S1 Table *S*. *mediterranea* UsnRNAs (Sheet SnRNA_identification) List of UsnRNA gene candidates identified by INFERNAL cmsearch and their RNAseq read counts. Genes present in multiple, identical copies are marked in the same colour. (Sheet SnRNA_classification) List of all UsnRNA gene candidates with >10 unique reads plus the U4atac candidate. Nomenclature according to sequence similarity and number of best-mapping reads.
(XLSX)

S2 Table Integrator complex homologs in *S*. *mediterranea*. List of putative *Smed ints* homologs identified by tBLASTx search in the dd_Smed_v6 transcriptome. The sequences of human, mouse and fruitfly *ints* were used as query. All hits with e-value < 10–5 are shown. N alignments indicates the number of sub-sequences producing hits. For *ints5* and *ints10*, putative homologs were identified from the Planmine blast annotation.
(XLSX)

S3 Table Expression of putative *ints* homologs in RNAseq data of FACS-sorted cells. The corresponding IDs in the to_Smed_v2 transcriptome, the respective normalized expression values and the original classification (SC = stem cell, X1-fraction; Prog = Progeny, X2-fraction; Tissues = Xins-fraction) are shown [41]. For *ints3*, the expression values (FPKM) were obtained from the Planmine annotation of transcript dd_Smed_v6_4426_0_1.
(XLSX)

S4 Table List of *ints* RNAi-induced splicing changes. Output of the vast-tools analysis. Column headers: **GENE**—Gene symbol of mammalian ortholog; **EVENT—**VAST-DB event ID; **COORD**—Genomic coordinate of the alternative sequence; **LENGTH**—Length of the alternative sequence; **FullCO**—Full set of genomic coordinates of the alternative splicing event; **COMPLEX**—Type of event; **dPSI *ints* RNAi**—Difference of inclusion levels (PSI *ints*—PSI GFP); **X1_GFP_c—**Estimated percent of sequence inclusion in sample GFP, Replicate c; **X1_GFP_c-Q—**Quality scores, and number of corrected inclusion and exclusion reads for sample GFP, Replicate c. The quality scores are encoded as follows: **A,B,C,D,E@F,G:** A—Read coverage based on actual reads; B—Read coverage based on corrected reads; C—Read coverage, based on uncorrected reads mapping only to the reference C1A, AC2 or C1C2 splice junctions; D—Imbalance of reads mapping to inclusion splice junctions; E—Complexity of the event; F&G—total number of reads, corrected for mappability, supporting inclusion (F) and exclusion (G). For a detailed description see: https://github.com/vastgroup/vast-tools#comparing-psis-between-samples. **Sheet Summary:** Numbers of identified splicing changes detected in *ints* RNAi (columns B&C), of those present in the VAST-DB and evaluated for the respective comparison (column D) and enrichment of exon skipping events compared with the respective event type (columns E&F).
(XLSX)

S5 Table DEseq2 analysis of X1-cell RNAseq data. Normalized read counts, moderated Log_2_ fold-change and statistical features as reported by DESeq2 (see Methods) for *ints3* and *ints9* RNAi in comparison to control RNAi. Sheet comparison Labbé et al 2012: Comparison of gene expression changes induced by *ints* RNAi to the transcripts differentially expressed between the FACS-fractions X1, X2 and Xin as defined in [41]. Up- and down-regulated transcripts relative to control were analyzed separately. P-values were calculated using a one-sided hypergeometric test.
(XLS)

S6 Table Primers and qRT-PCR probes used in this study. RNA polymerase promoter sequences are shown in lower case.
(XLSX)

S7 Table Treatment schedule. (XLSX)
