# Supplementary figures and images for: The Integrator complex regulates differential snRNA processing and fate of adult stem cells in the highly regenerative planarian Schmidtea mediterranea
Source: PLoS Genet. 2018 Dec 17;14(12):e1007828. doi: 10.1371/journal.pgen.1007828 (PMC6312358; doi:10.1371/journal.pgen.1007828)

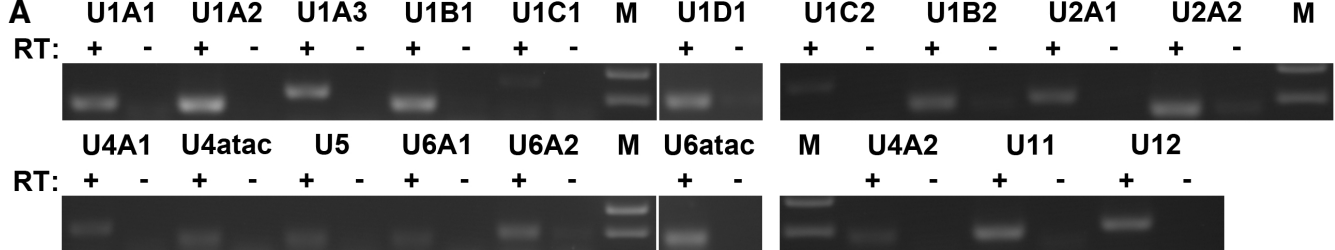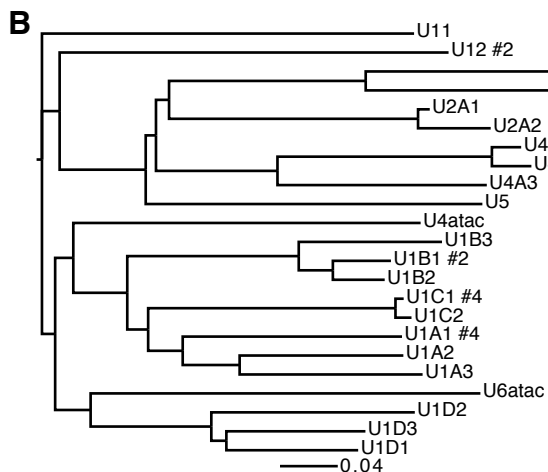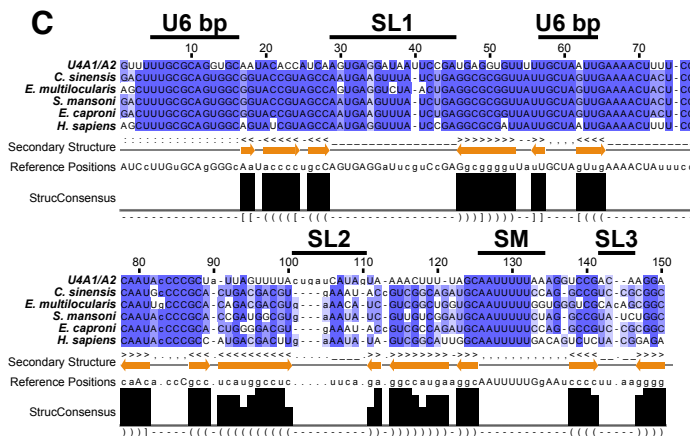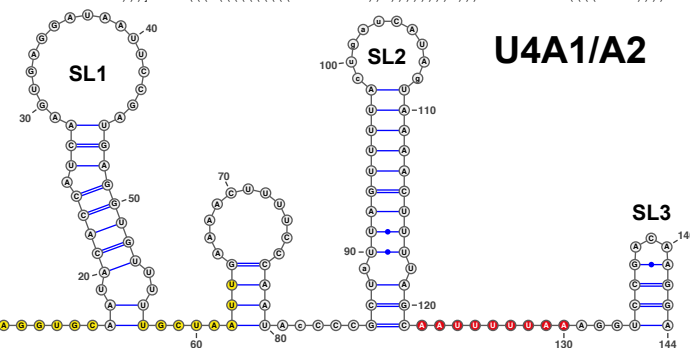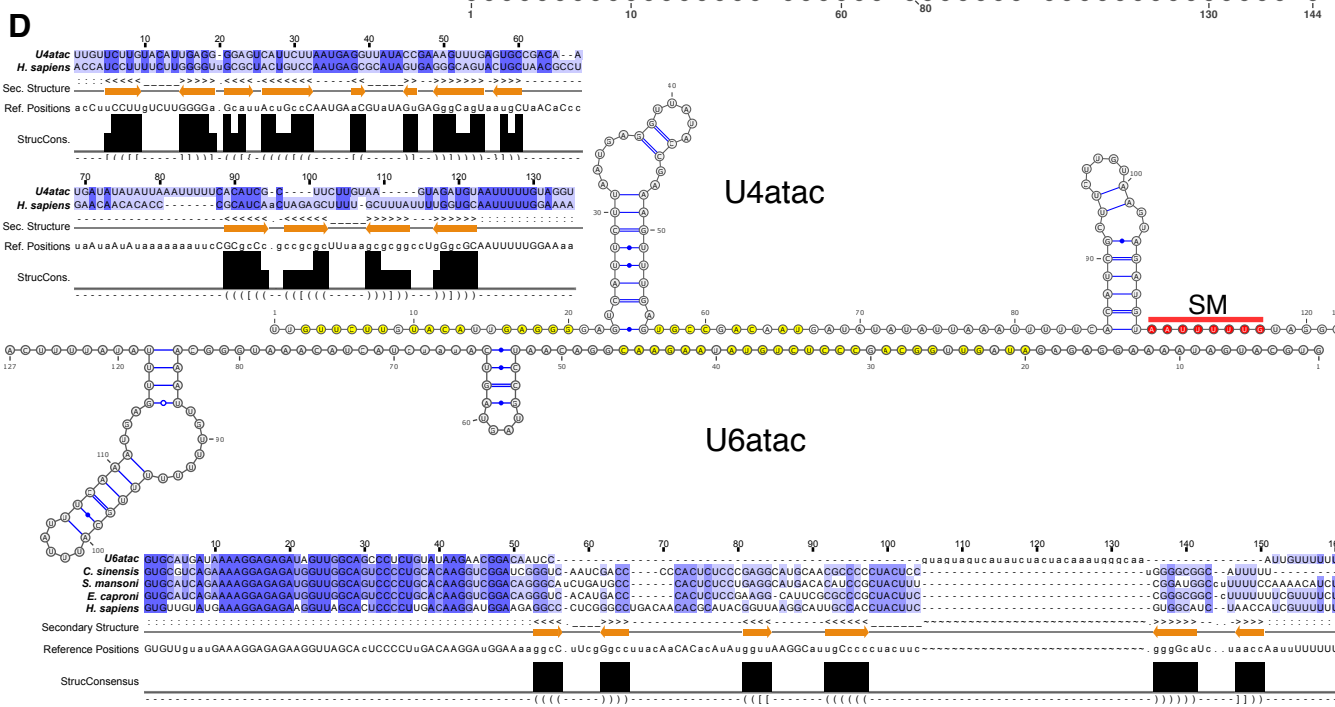

Supplement: S1 Fig — (A) Validation of UsnRNA expression. RT-PCR of all tested UsnRNAs produced bands of the expected size (M: molecular weight marker; 100 & 200 bp). Corresponding reactions without reverse transcriptase (-RT), which control for amplification from genomic DNA, had no or a significantly weaker band. (B) Clustering of validated UsnRNAs based on sequence similarity. # indicates the number of identical copies identified in the S. mediterranea genome [29]. (C+D) U4, U4atac and U6atac snRNA predicted secondary structures and sequence alignments to known homologs of other Platyhelminthes (Clonorchis sinensis, Schistosoma mansoni and Echinostoma caproni) and Homo sapiens. Primary sequence similarity is indicated in violet and secondary structure conservation (StrucConsensus) is shown below the alignments. Evolutionary conserved functional sequence elements are indicated: SM = SM protein binding site (red); U4-U6 or U4atac-U6atac base-pairing (yellow). (PDF) [file pgen.1007828.s001.pdf]

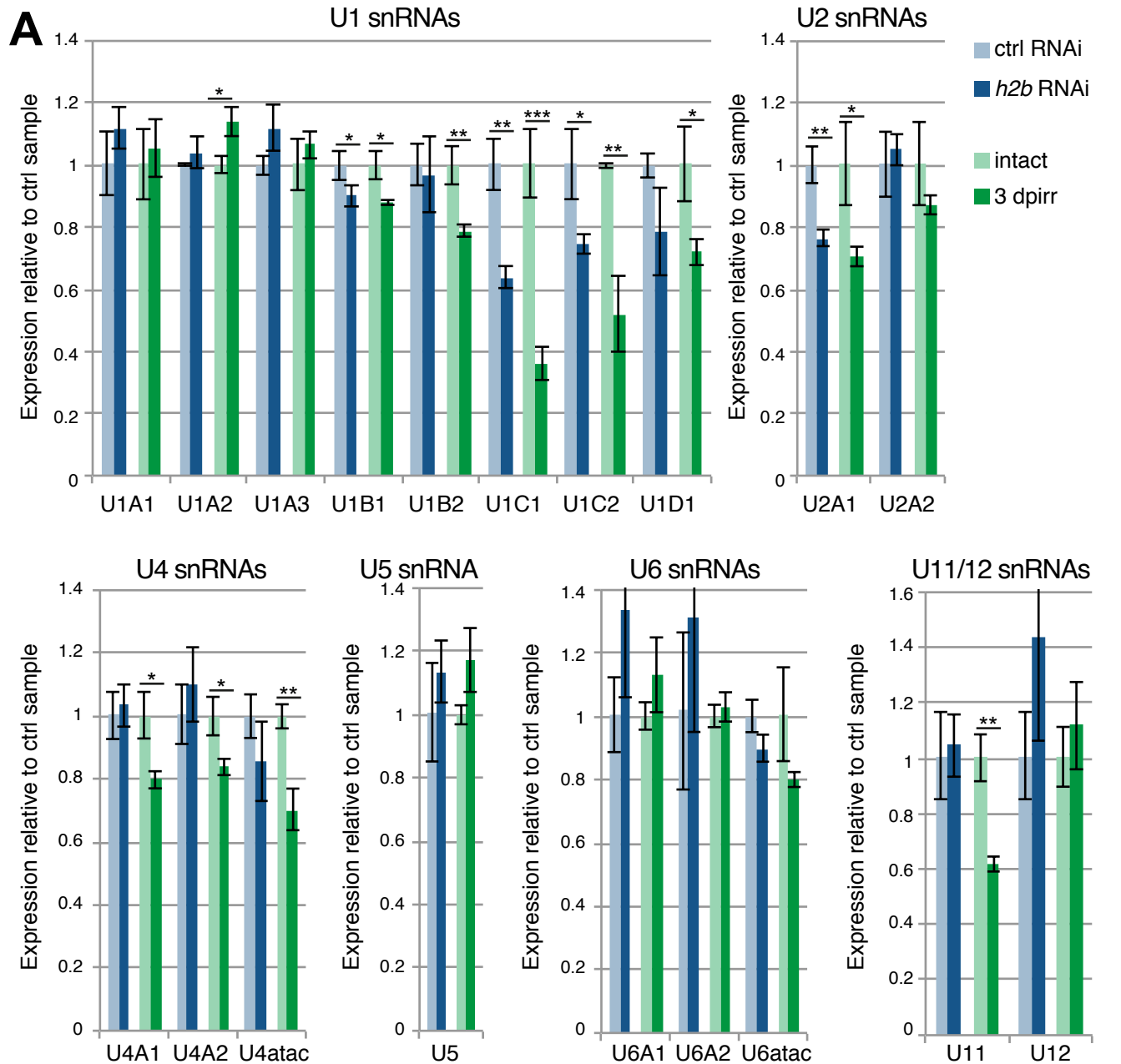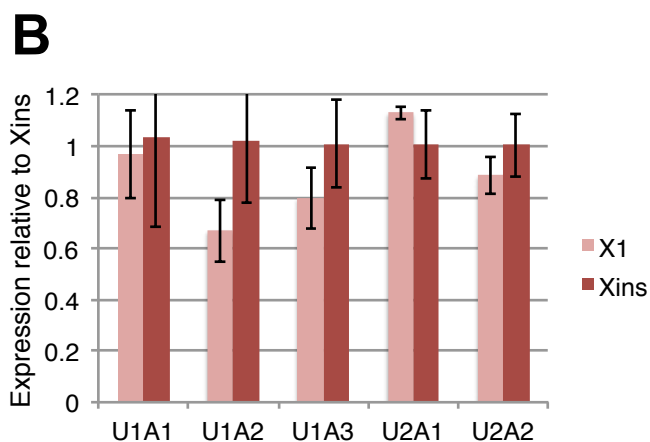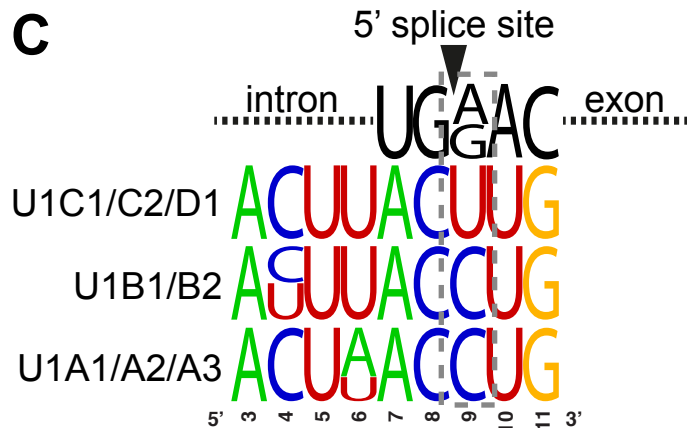

Supplement: S3 Fig — (A+B) UsnRNA variant expression in animals depleted of neoblasts by h2b RNAi (blue), by lethal irradiation (green) and in cell fractions isolated by FACS (red), quantified by qRT-PCR. Expression levels are the averages of three biological replicates, normalized to those of gapdh (two-sided t-test, * p<0.05, ** p<0.01, *** p<0.001). (C) Sequence logos of the 5’ splice site recognition motif of different U1 snRNA variants. The U1 variants with a high average neoblast-enrichment have an alternative base (C → U) annealing to the -1 position of the 5’splice site. (PDF) [file pgen.1007828.s003.pdf]

**A**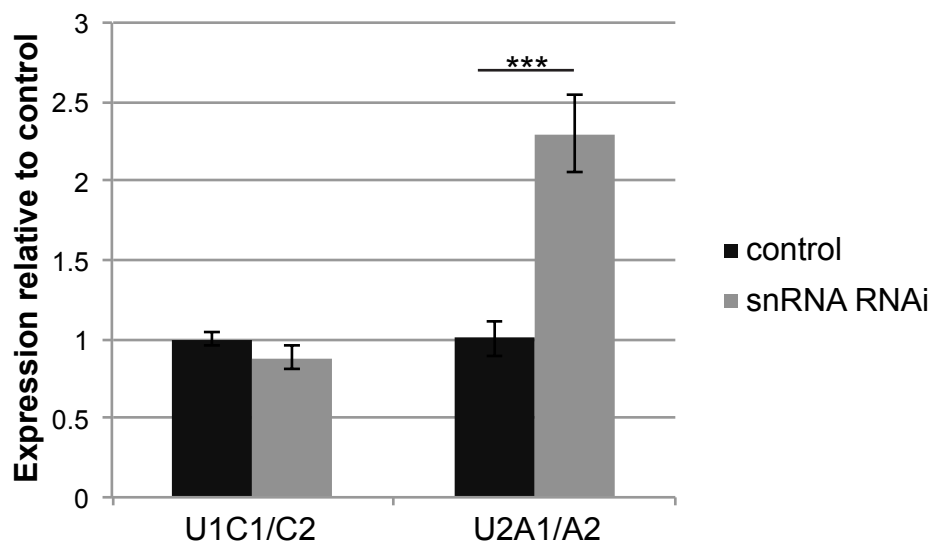**B**

U2A2 snRNA 3' end  
adapter sequence

A G T T T G G T C T G C T T G T T A G A T C G G A A G A G C A C A C A

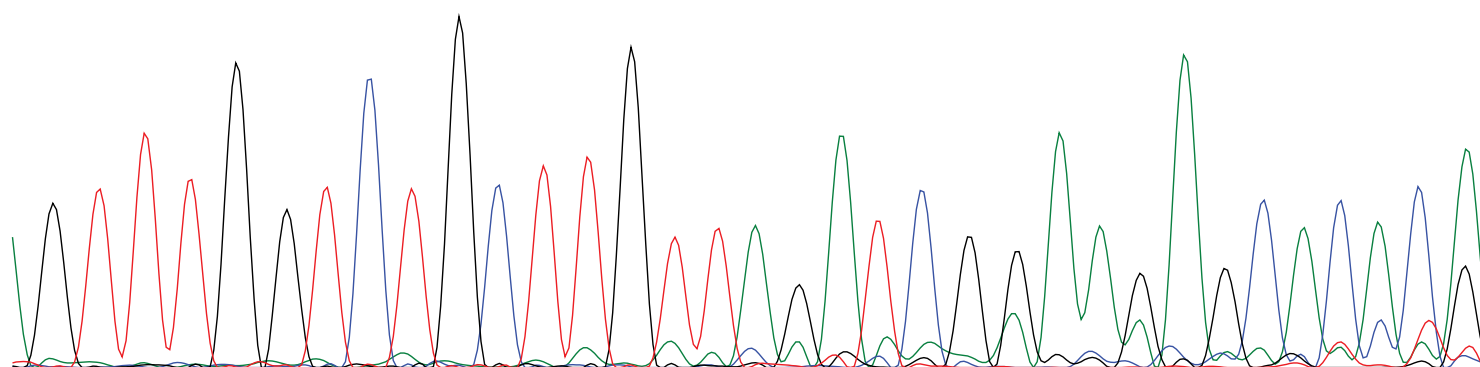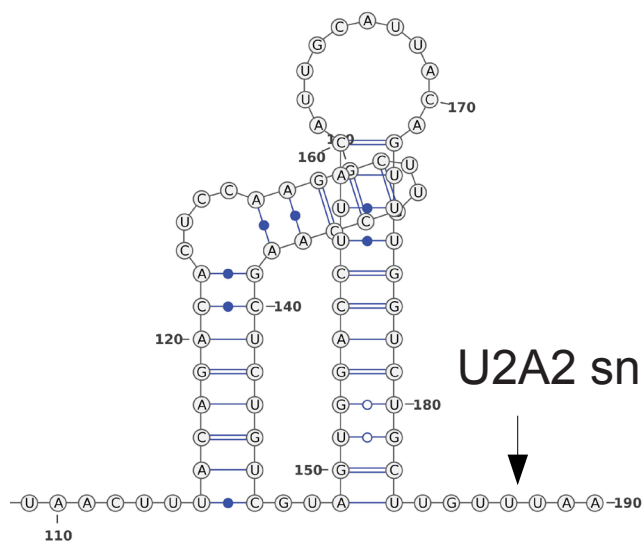

Supplement: S4 Fig — (A) Injection of dsRNAs targeting U1C or U2A snRNAs did not reduce their expression after 14 days of RNAi. Expression levels are the average of at least 3 biological replicates, normalized to those of gapdh (two-sided t-test, *** p<0.001). (B) Sanger sequence trace showing the 3’ end of U2A2 and the adapter of the RNAseq library from which it was amplified. The mature end is also indicated in the secondary structure model of U2A2 below. (PDF) [file pgen.1007828.s004.pdf]

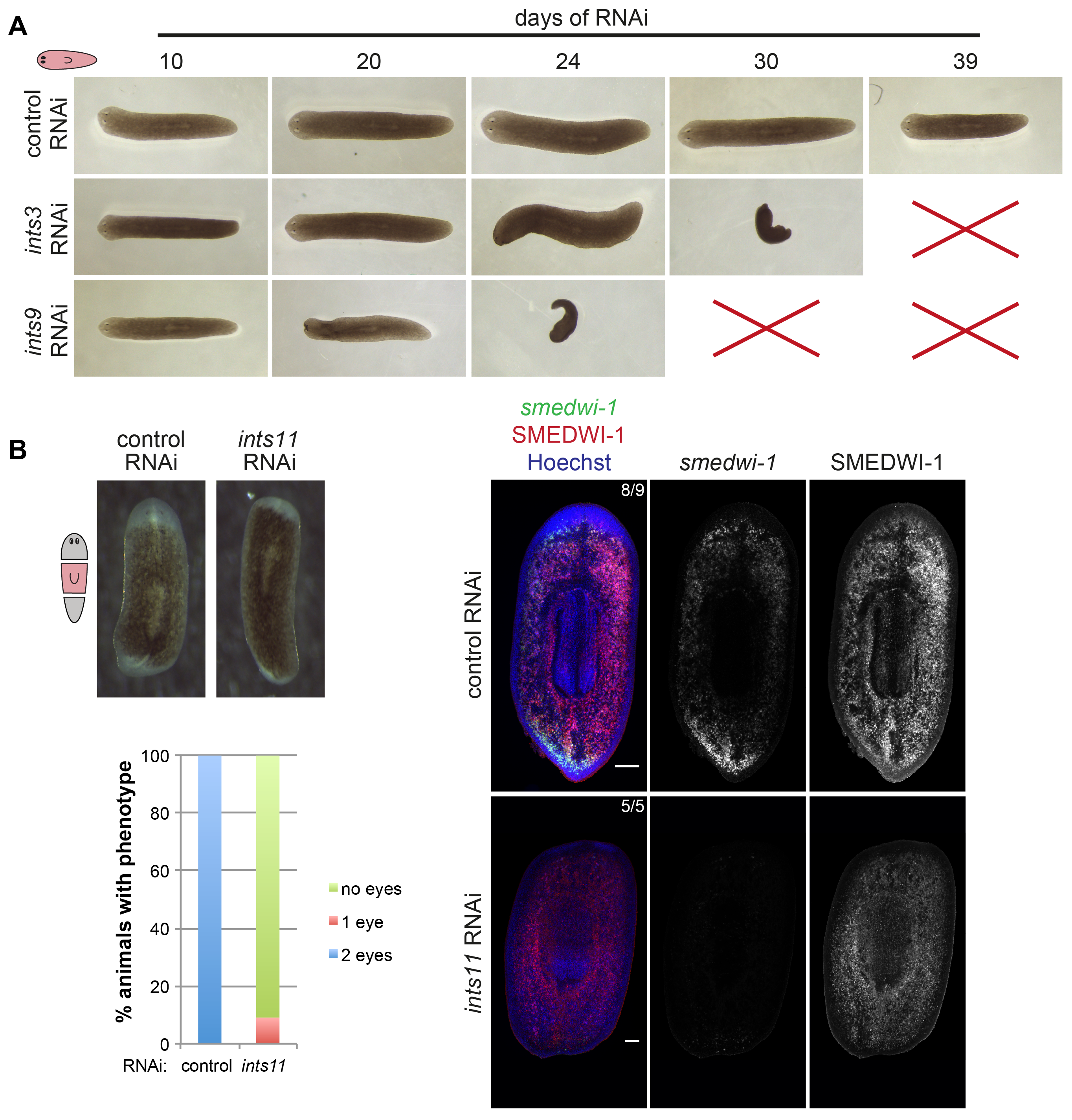

Supplement: S5 Fig — (A) Live microscopy images showing homeostatic RNAi animals at the indicated times from the first RNAi treatment (days of RNAi). ints RNAi animals developed head regression, ventral curling and eventually lysed. (n = 21, 2 independent experiments). (B) Depletion of the putative ints11 homolog pheno-copied the regeneration and neoblast maintenance defects induced by ints3 and ints9 RNAi. All images and data are from trunk fragments at 8 dpa (n > 10 for live images, 21 d RNAi, 1 experiment). Scale bar = 100 μm. (TIF) [file pgen.1007828.s005.tif]

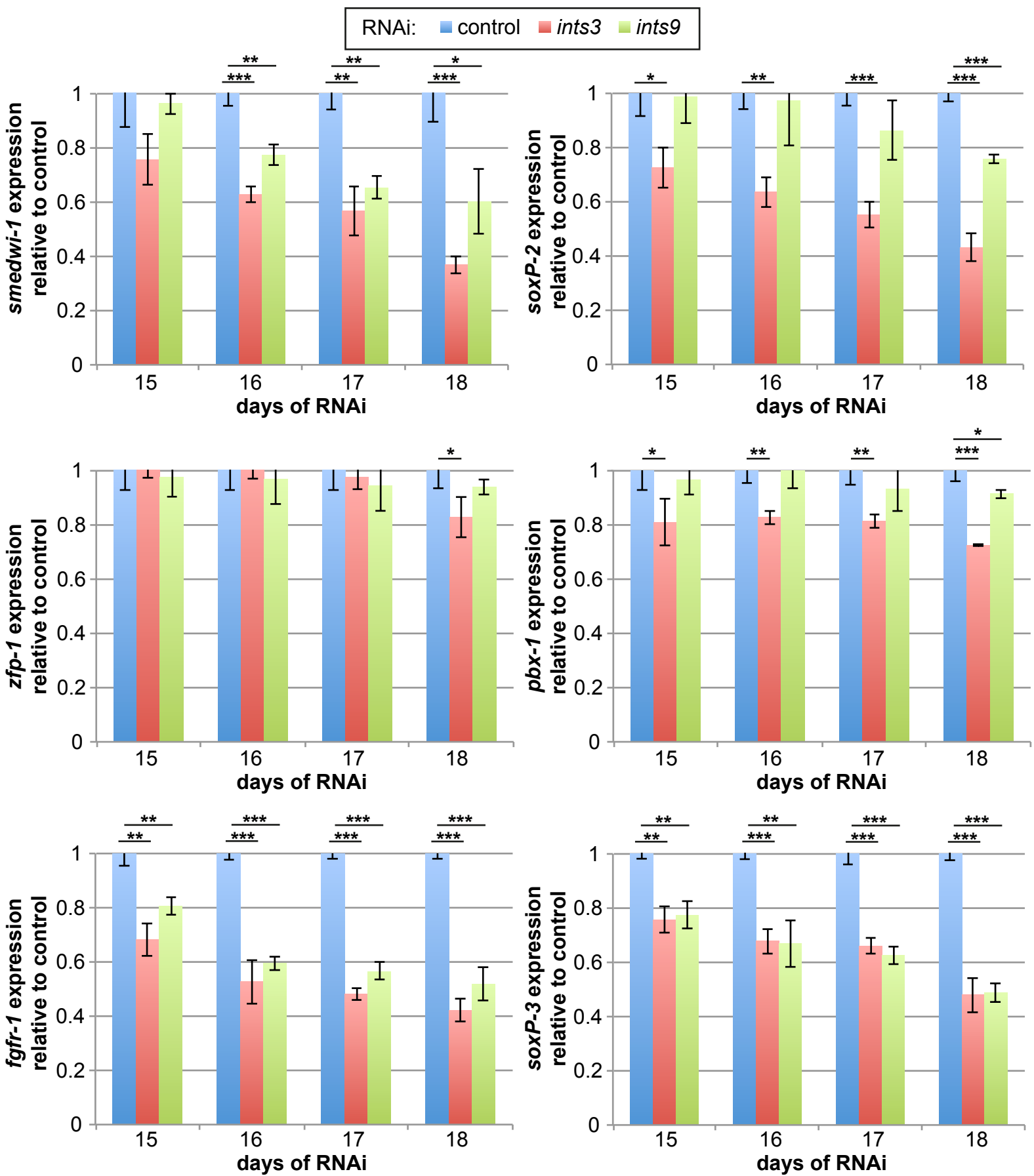

Supplement: S6 Fig — The zeta and sigma neoblast subclass markers were described in [46]. The expression levels are the averages of three biological replicates normalized to those of gapdh. (two-sided t-test, * p<0.05, ** p<0.01, *** p<0.001). (PDF) [file pgen.1007828.s006.pdf]

**A**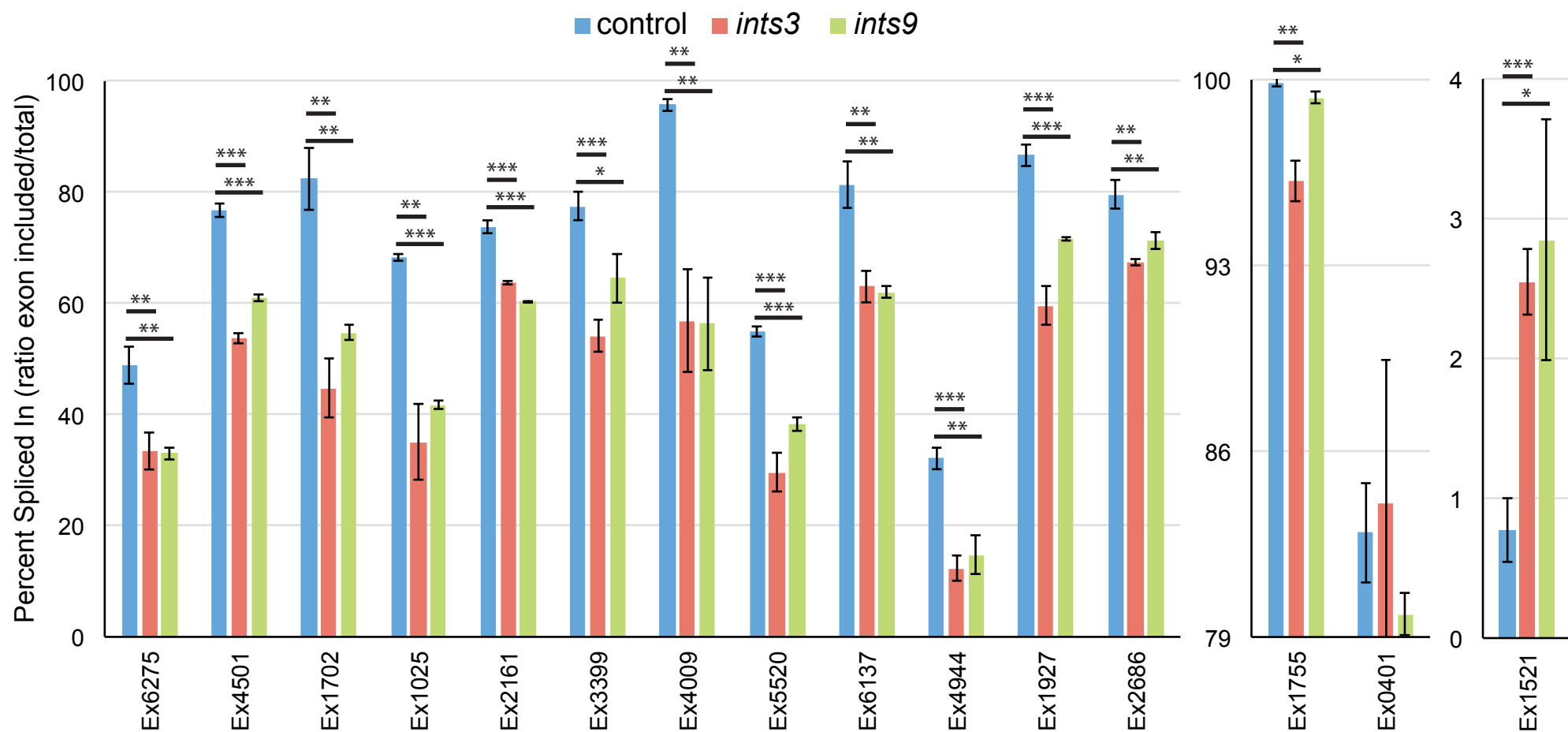**B**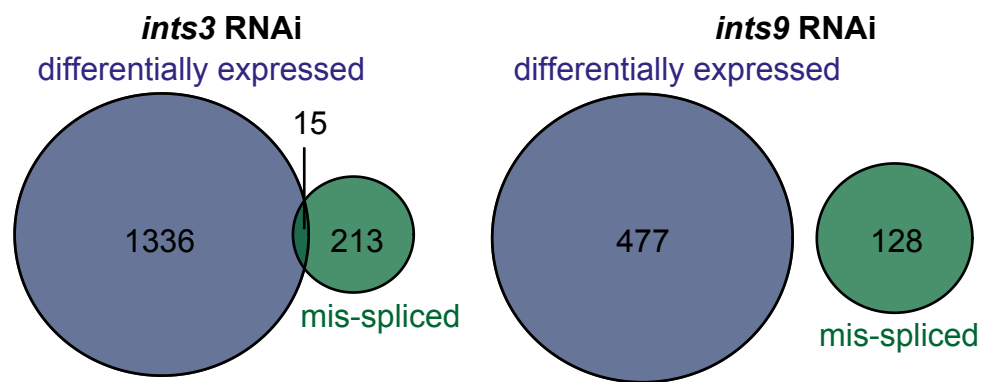**C**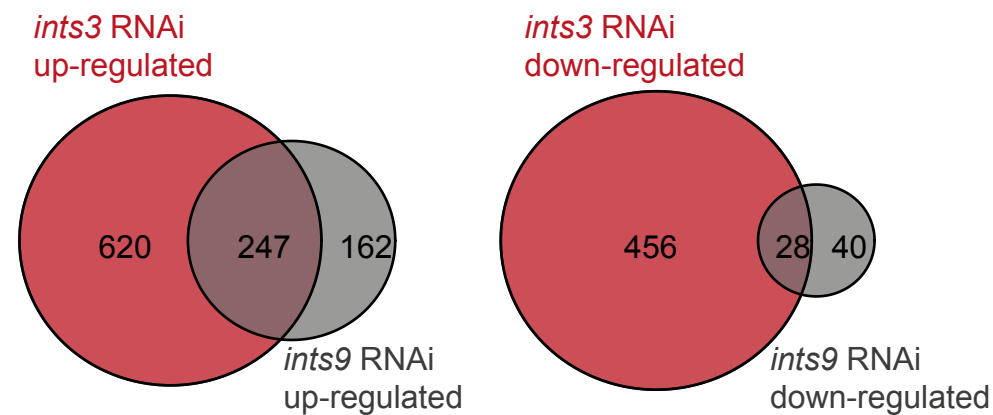

Supplement: S7 Fig — (A) Results of the RT-PCR validation of 15 alternative exon splicing events identified by RNAseq of ints RNAi X1 cells. The intensity of the gel bands (see Fig 4B for an example) representing the exon-included and the exon-excluded form were quantified and used to calculate the PSI value. 14 of the 15 events tested were significantly de-regulated in ints3 and ints9 RNAi X1 cells. The events IDs correspond to those in S4 Table (SmeEX(0)nYYYY → ExYYYY). (B, C) Venn-Diagrams showing that the vast majority of the detected splicing defects do not significantly affect the expression of the respective transcript and that a substantial number of gene expression changes are shared by ints3 and ints9 RNAi X1 cells. Thresholds for inclusion are the same as in Fig 5B and 5C. (PDF) [file pgen.1007828.s007.pdf]
