# Supplementary material for: The Integrator complex regulates differential snRNA processing and fate of adult stem cells in the highly regenerative planarian Schmidtea mediterranea
Source: PLoS Genet. 2018 Dec 17;14(12):e1007828. doi: 10.1371/journal.pgen.1007828 (PMC6312358; doi:10.1371/journal.pgen.1007828)
Supplement: S2 Fig — Representative predicted secondary structures and sequence alignments to their homologs of other Platyhelminthes (Clonorchis sinensis, Echinococcus multilocularis, Schistosoma mansoni and Echinostoma caproni) and Homo sapiens. Primary sequence similarity is indicated in violet and secondary structure conservation (StrucConsensus) is shown below the alignments. Evolutionary conserved functional sequence elements are indicated: ss = splice site recognition motif (green); SM = SM protein binding site (red); BPR = branch-point recognition sequence (blue); UsnRNA base-pairing sequence (yellow). (PDF) [file pgen.1007828.s002.pdf]

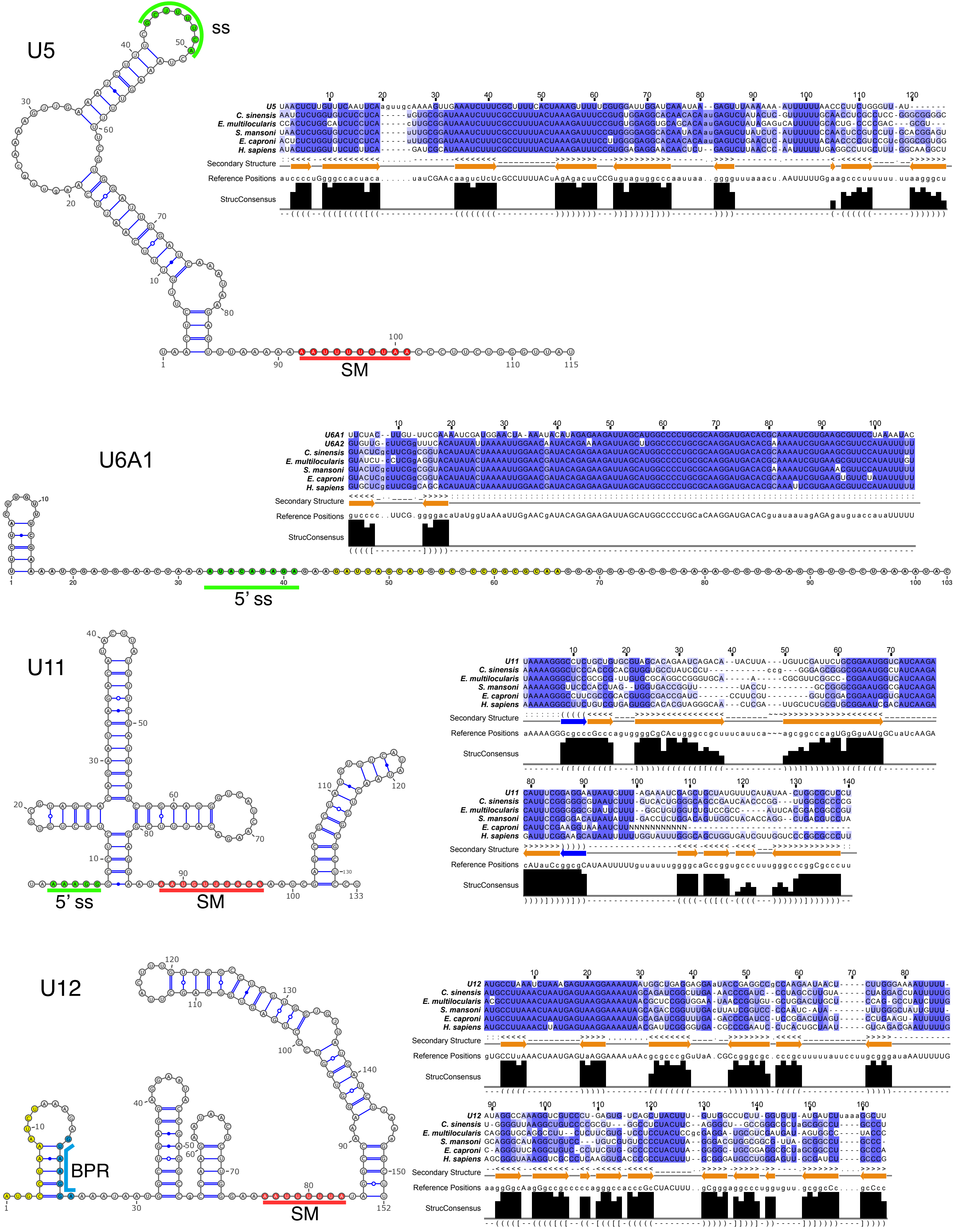

U5
ss
SM
U5
C. sinensis
E. multilocularis
S. mansoni
E. caproni
H. sapiens
Secondary Structure
Reference Positions
StrucConsensus
U6A1
5' ss
U6A1
U6A2
C. sinensis
E. multilocularis
S. mansoni
E. caproni
H. sapiens
Secondary Structure
Reference Positions
StrucConsensus
U11
5' ss
SM
U11
C. sinensis
E. multilocularis
S. mansoni
E. caproni
H. sapiens
Secondary Structure
Reference Positions
StrucConsensus
U12
BPR
SM
U12
C. sinensis
E. multilocularis
S. mansoni
E. caproni
H. sapiens
Secondary Structure
Reference Positions
StrucConsensus
