# Supplementary material for: The Integrator complex regulates differential snRNA processing and fate of adult stem cells in the highly regenerative planarian Schmidtea mediterranea
Source: PLoS Genet. 2018 Dec 17;14(12):e1007828. doi: 10.1371/journal.pgen.1007828 (PMC6312358; doi:10.1371/journal.pgen.1007828)
Supplement: S8 Fig — (A, B) Prediction of upstream and downstream splice site strength. (C) Determination of exon length. (D) GC content in affected exons. (E, F) Median length of down- and up-stream introns of affected exons. (G) Statistical significance (p-values) for analyses shown in A-F tested by Mann-Whitney-U-test. AS NC alternatively spliced, but not affected by ints RNAi; CR “cryptic” exons (PSI < 5); CS random subset of 1000 constitutively spliced exons; EX exons with ΔPSI < -15 in ints vs control RNAi; INC exons with ΔPSI > 15 in ints vs control RNAi; bp basepair; ss splice site (PDF) [file pgen.1007828.s008.pdf]

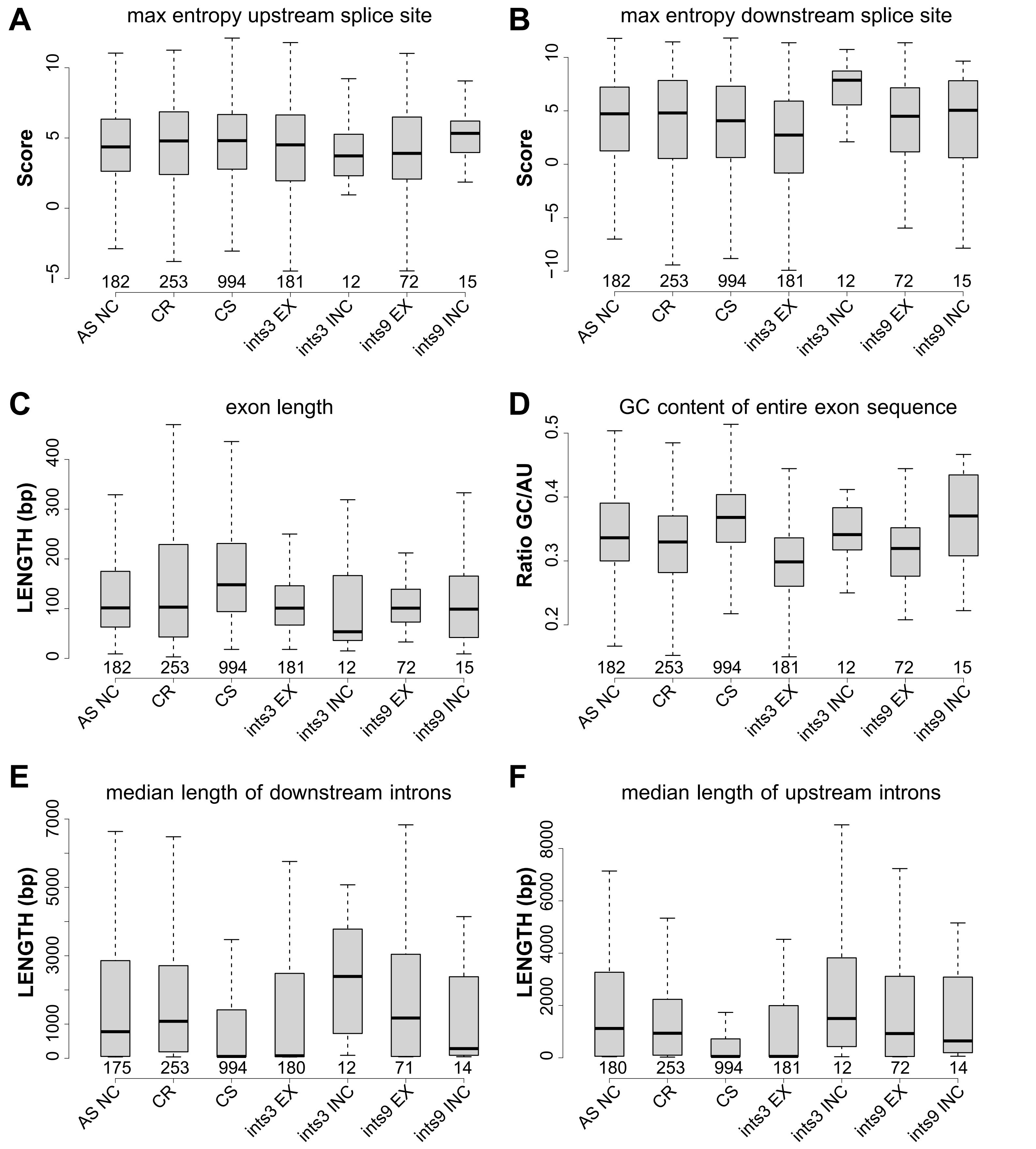

G

	max entropy upstream ss	max entropy downstream ss	exon length	GC content	median length upstream intron	median length downstream intron
ints3 EX vs AS NC	ns	1.55E-03	ns	3.15E-11	3.59E-06	9.12E-04
ints3 EX vs CR	ns	3.07E-04	ns	4.19E-05	4.03E-08	1.45E-07
ints3 EX vs CS	ns	2.31E-03	9.80E-15	3.91E-38	ns	ns
ints9 EX vs AS NC	ns	ns	ns	1.22E-03	ns	ns
ints9 EX vs CR	ns	ns	ns	ns	ns	ns
ints9 EX vs CS	ns	ns	6.03E-08	9.18E-12	1.01E-05	4.91E-06
